# CDK7–CDK11 axis in spliceosome regulation and pre-mRNA splicing

**DOI:** 10.1093/nar/gkaf1343

**Published:** 2025-12-22

**Authors:** Michal Rájecký, Pavla Gajdušková, Peter Maník, Milan Hluchý, Eva Hegedűsová, Karolína Kryštofová, David Potěšil, Petra Martinková, Zuzana Slabá, Prashant Khirsariya, Marek Šebesta, Kamil Paruch, Caroline C Friedel, Zbyněk Zdráhal, Dalibor Blazek

**Affiliations:** Central European Institute of Technology (CEITEC), Masaryk University, Brno 62500, Czech Republic; Central European Institute of Technology (CEITEC), Masaryk University, Brno 62500, Czech Republic; Central European Institute of Technology (CEITEC), Masaryk University, Brno 62500, Czech Republic; National Centre for Biomolecular Research, Faculty of Science, Masaryk University, Brno 62500, Czech Republic; Central European Institute of Technology (CEITEC), Masaryk University, Brno 62500, Czech Republic; Central European Institute of Technology (CEITEC), Masaryk University, Brno 62500, Czech Republic; Central European Institute of Technology (CEITEC), Masaryk University, Brno 62500, Czech Republic; Central European Institute of Technology (CEITEC), Masaryk University, Brno 62500, Czech Republic; Central European Institute of Technology (CEITEC), Masaryk University, Brno 62500, Czech Republic; National Centre for Biomolecular Research, Faculty of Science, Masaryk University, Brno 62500, Czech Republic; Central European Institute of Technology (CEITEC), Masaryk University, Brno 62500, Czech Republic; Department of Chemistry, Masaryk University, Brno 62500, Czech Republic; International Clinical Research Center, St. Anne’s University Hospital, Brno 62500, Czech Republic; Central European Institute of Technology (CEITEC), Masaryk University, Brno 62500, Czech Republic; Department of Chemistry, Masaryk University, Brno 62500, Czech Republic; International Clinical Research Center, St. Anne’s University Hospital, Brno 62500, Czech Republic; Institute for Informatics, LMU Munich, Munich 80333, Germany; Central European Institute of Technology (CEITEC), Masaryk University, Brno 62500, Czech Republic; National Centre for Biomolecular Research, Faculty of Science, Masaryk University, Brno 62500, Czech Republic; Central European Institute of Technology (CEITEC), Masaryk University, Brno 62500, Czech Republic

## Abstract

Cyclin-dependent kinase 11 (CDK11) is essential for the regulation of pre-mRNA splicing via phosphorylation of the core spliceosome component SF3B1. This phosphorylation is a marker of the catalytically active spliceosomes; thus, it is important to identify the mechanisms that regulate CDK11 itself. Here, we report that a small subset of CDK11 is phosphorylated on the activation T-loop threonine 595 (Thr595) and is associated with the activated spliceosome on chromatin in gene bodies. Mutational analyses revealed that Thr595 is essential for the formation of the active CDK11 complex with cyclin L and SAP30BP. CDK11 transiently associates with CDK7, a transcriptional kinase that also promotes the activation of other CDKs. Inhibition of CDK7 initially decreases transcription, but longer durations of inhibition lead to production of unspliced pre-mRNAs. The onset of the CDK7-mediated splicing defect correlates with the sequential dephosphorylation of CDK11 Thr595 and SF3B1. SILAC-based phosphoproteomics upon brief CDK11 inhibition identified SF3B1, CDC5L, and ESS2 as CDK11 substrates, which overlap with the previously identified CDK7 substrates in the spliceosome. In summary, our study suggests that CDK7 likely acts via CDK11 Thr595 phosphorylation to regulate pre-mRNA splicing in cells. The identification of additional CDK11 substrates points to its broader role in spliceosome regulation.

## Graphical Abstract

**Graphical Abstract:**
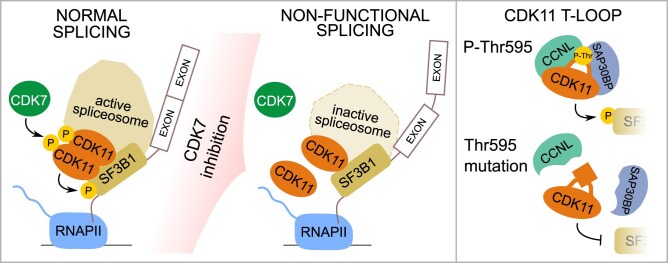

## Introduction

Cyclin-dependent kinases (CDKs) are serine (Ser)/threonine (Thr) kinases that play key roles in regulating cell cycle transitions (CDK1, CDK2, and CDK4/6), transcription (CDK7, CDK8, CDK9, and CDK12), and splicing (CDK11) [1–3]. To be catalytically functional, CDKs need to be activated, which typically occurs in two steps: the binding of a cyclin cofactor, and the phosphorylation of a canonical Thr in the activation T-loop of the CDK [4]. Cyclin binding induces a conformational change in the CDK, partially exposing its active site and providing most of the kinase’s stimulation [5]. Full activation usually requires phosphorylation of Thr in the T-loop, which alters its shape and enhances the active site’s ability to bind substrates [6, 7].

CDK7 acts as the CDK-activating kinase (CAK) as part of a trimeric CAK complex with cyclin H (CCNH) and MAT1 [2, 8]. It phosphorylates the T-loop and activates both cell cycle (CDK1, CDK2, and CDK4/6) and transcription (CDK9 and CDK12) CDKs, making it a master regulator of transcription and cell cycle progression [9–12]. CDK7 also participates in forming the transcription preinitiation complex, as part of the 10-subunit TFIIH complex (7 subunits of core complex and 3 subunits of CAK) [13, 14] where CDK7 phosphorylates the C-terminal domain (CTD) of RNA polymerase II (RNAPII) on serine 5 and 7 (Ser5 and Ser7) [15– 17]. This phosphorylation directs the release of Mediator and initiation factors from RNAPII and facilitates its promoter escape [10, 18]. In metazoans, this generally involves the transcription of ∼30–80 nucleotides downstream of the transcription start site (TSS), before the recruitment of pausing factors (NELF, DSIF) leads to promoter-proximal pausing, another regulatory step in RNAPII transcription [10, 18, 19]. CDK7 inhibition prevents the RNAPII-CTD-mediated release of Mediator and initiation factors, which forestalls the recruitment of pausing factors [10, 18, 20, 21]. Despite this, RNAPII can still slowly elongate, bypassing promoter-proximal pausing. Further downstream, other transcriptional CDKs compensate for CDK7, which leads to phosphorylation of the CTD and the replacement of Mediator and initiation factors with elongation complexes, and a switch to normal elongation [11, 18, 22].

Recent studies have identified widespread changes in pre-mRNA splicing upon CDK7 inhibition [11, 23, 24]. The splicing deficiencies caused by the selective CDK7 inhibitor SY-351 share some similarities with those elicited by the general splicing inhibitor pladienolide B. However, the mechanism remains unclear. The identification of CDK7 substrates suggested that CDK7 directly phosphorylates SF3B1 and other core spliceosome components, including CDC5L and ESS2 [11].

Given that CDK7 inhibitors such as THZ1 and SY-1365 selectively target cancer cells with elevated transcription [25, 26] and that other covalent inhibitors (SY-5609, Q901, and XL102) are in clinical trials [2, 27, 28], understanding the mechanisms and dynamics of CDK7-mediated regulation of splicing and transcription is of high importance.

The spliceosome is a large, multi-protein/small nuclear RNA (snRNA) complex that assembles, primarily co-transcriptionally, on exon–intron boundaries of nascent RNA to excise introns and form mature RNA (mRNA). The spliceosome’s lifecycle, which accompanies pre-mRNA splicing, involves assembly, activation, a two-step splicing catalysis, and disassembly [29–31]. SF3B1, a core component of the spliceosome, is phosphorylated by CDK11 [3, 32]. The hyperphosphorylation of SF3B1’s intrinsically disordered region (IDR) N-terminus is essential for the formation of the activated spliceosome, and is also present in the catalytically active spliceosome [3, 32–35]. Phosphorylated SF3B1 (P-SF3B1) represents only 10%–15% of the total SF3B1 in cells, and is associated with chromatin as part of spliceosomes engaged in co-transcriptional splicing [3, 32, 35–38]. The IDR contains 28 repeats of Thr/Pro CDK-consensus sites and additional Ser and Thr residues [3]. SF3B1 hyperphosphorylation is blocked rapidly (within minutes) by the CDK11 inhibitor OTS964, which suppresses spliceosome activation and pre-mRNA splicing both in cells and *in vitro*, providing strong evidence for CDK11’s direct role in spliceosome activation [32].

The *CDK11* gene is duplicated in the human genome, with two almost identical isoforms called *CDK11A* and *CDK11B* [39]. They produce the 110 kDa form of CDK11 that is abundant and ubiquitously expressed in cells and contains a kinase domain and disordered N- and C-termini [3]. A shorter 58 kDa isoform of CDK11 (CDK11^58^) has also been reported. It corresponds to amino acids 344–782 of CDK11B and is expressed exclusively during the G2/M phase [40], but it has not been detected in many cell lines [41, 42]. CDK11 binds one of the cyclin L (CCNL) proteins, CCNL1 or CCNL2 [43], which are functionally redundant [44]. The complex is stabilized by SAP30BP, which is essential for CDK11 kinase activity [33]. In addition to the fundamental roles of CDK11 in regulating normal gene expression [32, 45, 46], the kinase is an essential candidate gene for the proliferation of various cancers [47, 48]. Consistent with this, OTS964 exhibited anticancer activity in an animal model [49] and inhibits the proliferation of many cancer cell lines [32, 50, 51]. Therefore, characterizing the upstream pathways that impact CDK11 activity and identifying CDK11 substrates is of significant interest [3].

We used proximity proteomics to find regulators of CDK11 and identified CDK7 as a potential candidate. To dissect CDK7´s roles in transcription and splicing and to separate its primary and secondary effects on splicing, we used highly selective inhibitors, time-course experiments, phospho-specific antibodies and an *in vitro* splicing assay. By monitoring changes in the dynamics of (de)phosphorylation of Thr595 in CDK11’s T-loop and SF3B1, we obtained the first evidence on order of events and a potential CDK11-dependent mechanism via which CDK7 acts on the spliceosome. CDK7 kinase activity is needed for the formation of the activated spliceosome which carries CDK11 phosphorylated on Thr595. The time-course experiments and identification of additional CDK11 substrates that overlap with the ones recently found for CDK7 in the spliceosome [11] suggest that CDK7 acts indirectly via CDK11 to regulate pre-mRNA splicing.

## Materials and methods

### Synthesis of SY-351

SY-351 was prepared by synthesis that included modified final steps of the route published previously [26]. See Supplementary methods for detailed procedure.

### Plasmids, siRNAs, and cell lines

The list of plasmids, small interfering RNAs (siRNAs), and cell lines used in the study is in Supplementary Table 5, Supplementary Table 6, and Supplementary Table 7, respectively.

### Immunoprecipitation after inhibition of CDK7

Three 15-cm dishes of HCT116 cells at 75% confluency were inhibited with 50 nM SY-351 for 4 h. Dynabeads protein G (30 μl, Thermo Fisher Scientific, 10009D) were washed 3 × with 1 ml of HEPES lysis buffer (20 mM HEPES, pH 7.4, 100 mM KCl, and 0.5% Triton X-100), split into two tubes, and incubated for 2 h rotating at 4°C with 2 μg of either IgG (Proteintech, 30000-0-AP) or CDK11 (Abcam, ab19393) antibodies. Cells were harvested, pooled, washed 2× with ice-cold 10 mM sodium phosphate buffer, 154 mM sodium chloride, pH 7.4 (Phosphate Buffered Saline (PBS)) with 1000 × *g*/4°C/3 min centrifugation in between and lysed for 10 min in 1 ml of lysis buffer + Phosphatase Inhibitor Cocktail 3 (Sigma, P0044). Samples were sonicated on QSonica Q55A (5/64 probe) using 3 × 7 s pulse at 0.3 amplitude. Samples were centrifuged 10 000 × *g*/4°C/10 min, supernatant was transferred into new tubes, and 50 μl of it was saved as input. Dynabeads protein G were washed 3× with 1 ml of lysis buffer to remove unbound antibodies, and then supernatant was split in between both tubes with bound antibodies and incubated rotating for 1.5 h at 4°C. Dynabeads protein G were washed 3× with 1 ml of lysis buffer, dispersed in 30 μl of 3 × Laemmli buffer [180 mM Tris buffer, pH 6.8, 6% SDS, 30% glycerol, 15% β-mercaptoethanol (Sigma, M3148), 0.06% bromophenol blue (Acros Organics, 151340250)], and heated for 3 min at 95°C. Experiment was run in two biological replicates.

### Western blotting

Standard electrophoresis and wet transfer procedures were followed. Antibodies are listed in Supplementary Table S3. All antibodies used for detection of phospho-specific epitopes were diluted in 20 mM Tris buffer, 150 mM NaCl, and 0.1% Tween-20, pH 7.5 (TBS-T) supplemented with 5% bovine serum albumin (BSA, GE Healthcare, K41-001), which was also used for blocking of nitrocellulose membranes (Sigma, GE10600008). For detection of other proteins, PBS/0.1% Tween-20 (PBS-T) with 5% low-fat milk was used.

### Production of phosphospecific CDK11 antibodies

Rabbit CDK11 phosphospecific T-loop antibodies raised against phosphopeptides CPLKAY(Tp)PVVVTL (Ab1, Ab2) or CGSPLKAY(Tp)PVV (Ab3) were produced by Moravian Biotechnology (Czech Republic). Affinity-purified fractions were delivered for our own validation.

### Validation of antibodies: siRNA-mediated knockdown

HCT116 cells were plated in a six-well plate at 30% confluency for 3 h before transfection. Cells were transfected with siRNA at a final concentration of 10 nM using Lipofectamine RNAiMax (Thermo Fisher Scientific, 13778-150), according to manufacturer’s instructions. Briefly, 2.5 μl of siRNA (10 μM stock solution) was diluted into 250 μl of Opti-MEM (Thermo Fisher Scientific, 31985-070) and mixed together with 5 μl of lipofectamine diluted into 250 μl of Opti-MEM. After 15 min, the mixture was added drop-wise onto the cultured cells in 2 ml of medium. If larger plates were used for transfections, the amounts of reagents were scaled up proportionally. Cells were transfected with nontargeting control siRNA-A (Santa Cruz, sc-37007) or CDK11-targeting siRNAs (Sigma, SASI_WI_00 000 026 or Santa Cruz, sc-37591) for 40–42 h. Experiment was run in three biological replicates.

### Validation of antibodies: phosphatase treatment

One 15-cm dish of HCT116 cells at 40% confluency was harvested, centrifuged 1000 × *g*/4°C/3 min, washed 2× with ice-cold PBS, and lysed for 10 min in 200 μl of HEPES lysis buffer. Samples were sonicated on QSonica Q55A (5/64 probe) using 7 × 0.25 s pulse at 0.3 amplitude and lysed another 10 min. Samples were centrifuged 10 000 × *g*/4°C/10 min, supernatant was split into two tubes with addition of either 15 μl of FastAP and 5 μl of 10× FastAP buffer (Thermo Fisher, EF0654) or 1 μl of Phosphatase Inhibitor Cocktail 3 (Sigma, P0044) and 20 μl of HEPES lysis buffer. Samples were incubated 1 h at 25°C with shaking at 300 rpm. Then, 60 μl of 3 × Laemmli buffer was added and samples were heated at 95°C for 3 min. Experiment was run in three biological replicates.

### Inhibition of CDK7^AS/AS^ HCT116 cells with 3MB-PP1

HCT116 CDK7^AS/AS^ cell line was a gift from R. Fisher [9]. Cells were treated with dimethyl sulfoxide (DMSO) or 40 μM 3MB-PP1 (Merck, 529582) for 4 h. Cells were harvested, washed 2× in PBS, and lysed in 50 μl of RIPA buffer (50 mM Tris buffer, pH 8, 150 mM NaCl, 5 mM EDTA, 1% NP-40, 0.5% sodium deoxycholate, 0.1% SDS, and Protease Inhibitor Cocktail, Sigma, P8340) + Phosphatase Inhibitor Cocktail 3 (Sigma, P0044). Samples were sonicated on QSonica Q55A (5/64 probe) using 7 × 0.25 s pulse at 0.25 amplitude and centrifuged 10 000 × *g*/4°C/10 min. Protein concentration in supernatant was equalized using bicinchoninic acid (BCA) assay (Thermo Fisher, 23225), and samples were heated at 95°C for 3 min in 3 × Laemmli buffer. Experiment was run in three biological replicates.

### Identification of CDK11^220^ in cells with stably integrated inducible flag-tagged CDK11

Two 10-cm dishes of HCT116 Flp-In cell line with stably integrated pcDNA5/FRT/TO CDK11 5′: 3xFLAG plasmid were induced overnight with 1 μg/ml of doxycycline (Sigma, D3072). Two dishes were left uninduced as a control. Cells were harvested, washed 2 × in PBS and lysed in 200 μl of RIPA buffer. Samples were sonicated on QSonica Q55A (5/64 probe) using 15 × 0.25 s pulse at 0.3 amplitude and centrifuged 10 000 × *g*/4°C/10 min. Supernatant was mixed with 3 × Laemmli buffer and heated at 95°C for 3 min. Experiment was run in two biological replicates.

### CDK11 dimerization assay

HEK293 cells were co-transfected with 6 μg of Myc-CDK11 plasmids and 6 μg of plasmid expressing indicated Flag-CDK11 constructs (Supplementary Fig. S2D and E) using polyethylenimine (PEI) (Poly sciences Inc., 24765). Medium was changed 3 h after transfection. Cells were collected 48 h after transfection, washed 2× with PBS, and lysed in lysis buffer (20 mM HEPES, pH 7.9, 150 mM KCl, 0.2% NP-40, 15% glycerol, 1 mM dithiothreitol (DTT), and Protease Inhibitor Cocktail, Sigma, P8340). After centrifugation at 10 000 × *g*/4°C/10 min equal amounts of supernatants were incubated with 15 μl of packed Flag agarose M2 affinity beads (Sigma, A2220) and rotated for 2 h at 4°C. Samples were washed 3 × with lysis buffer (rotated for 5 min at 4°C during each wash). After the last wash, remaining buffer was carefully removed and samples were heated in 50 μl of 3 × Laemmli buffer at 95°C for 3 min. Experiment was run in two biological replicates.

### Determination of CDK11^58^ expression in asynchronous and cell cycle-synchronized cells

To check expression of endogenous CDK11^58^, HCT116 cells were transfected with siRNA targeting CDK11 (Sigma, SASI_WI_00 000 027, Sigma, SASI_WI_00 000 026 or Santa Cruz, sc-37591) at final concentration of 10 nM using Lipofectamine RNAiMax (Thermo Fisher Scientific, 13778-150). Control samples were transfected with non-targeting control siRNA-A (Santa Cruz, sc-37007). Cells were harvested after 36 h. PBS-washed cell pellets were lysed with RIPA buffer, sonicated (5 × 1 s, amplitude 0.30, using 5/64 probe, QSonica Q55A) and centrifuged 10 000 × *g*/4°C/10 min. Protein concentration was equalized using BCA assay (Thermo Fisher, 23225), samples were mixed with 3 × Laemmli sample buffer and heated at 95°C for 2 min.

HCT116 cells were synchronized using a double-thymidine block method to check CDK11^58^ levels during cell cycle. Briefly, cells were blocked with 2 mM thymidine (Sigma, T1895) for 16 h, washed 2 × with PBS, released into fresh medium for 8 h, and blocked again with 2 mM thymidine for 16 h. Cells were released into fresh medium and collected at 2-h intervals. Lysis was done as described above. Experiment was run in two biological replicates.

### BioID and mass spectrometry identification of proteins

BioID was performed as described before [52]. Briefly, HEK293 cell line with integrated FRT/TO 5′ BirA-CDK11 3′ 3 × Flag plasmid and control cell line HEK293 FRT/TO 5′ 3 × Flag-BirA were induced for 24 h by 2 μg/ml of doxycycline so the expression of CDK11 is similar to endogenous level. Medium was changed for one containing 50 μM biotin (B4501, Sigma) and cells were incubated for 24 h. Cells were washed twice with 5 ml of PBS, harvested, and lysed in 1 ml of lysis buffer [50 mM Tris buffer, pH 7.4, 500 mM NaCl, 0.2% SDS, Protease inhibitor cocktail (P8340, Sigma), and 1 mM DTT] by passing through 0.8 × 40 mm needle twice and then through 21G × 1½” (insulin type) syringe three times. Triton X-100 was added to final concentration of 2% and lysate was passed through 0.8 × 40 mm needle 2‒3 times. Samples were sonicated 4 × 7 s, at amplitude 0.85 (QSonica Q55A, 5/64 probe). Addition of 2.2 ml of 50 mM Tris–HCl, pH 7.4 was followed by passing through the 0.8 × 40 mm needle (2‒3 times) and sonication 3 × 7 s, at amplitude 0.85. Samples were centrifuged 16 500 × *g*/4°C/10 min. About 1/12th of sample was kept for western blotting and the rest was mixed with 300 μl of Dynabeads MyOne Streptavidin C1 (65001, Thermo Fisher Scientific) pre-washed with lysis buffer. Samples were incubated overnight rotating at 4°C. Samples were washed twice with 2% SDS, once with 50 mM HEPES, pH 7.5, 500 mM NaCl, 1 mM EDTA, 0.1% sodium deoxycholate, 1% Triton X-100, once with 10 mM Tris–HCl, pH 7.4, 250 mM LiCl, 1 mM EDTA, 0.5% sodium deoxycholate, 0.5% NP-40, and resuspended in 1.5 ml of 50 mM Tris–HCl, pH 7.4. A 1/10th of samples was used for western blotting and the rest was used for MS.

Following IP washes, bead-bound protein complexes were digested directly on beads including proteins reduction (50 mM DTT, 30 min at 37°C) and alkylation [100 mM iodoacetic acid (IAA), 30 min in dark at laboratory temperature; quenched using 50 mM DTT for 30 min at 37°C] by addition of 1 μg of trypsin (sequencing grade, Promega) in 50 mM NaHCO_3_ buffer. Beads were gently tapped to ensure even suspension of trypsin solution and incubated at 37°C with mild agitation for 14 h. Peptide solutions were transferred into the LC-MS vial by 2.5% formic acid (FA) in 50% acetonitrile (ACN) and 100% ACN with addition of polyethylene glycol (PEG) (20 000, final concentration 0.001%) and concentrated in a SpeedVac concentrator (Thermo Fisher Scientific) to get rid of the ACN prior the LC-MS analyses [53]. LC-MS/MS analyses of all peptide mixtures were done using RSLCnano system (SRD-3400, NCS-3500RS CAP, WPS-3000 TPL RS) connected to Orbitrap Elite hybrid spectrometer (Thermo Fisher Scientific). Prior to LC separation, tryptic digests were online concentrated and desalted using trapping column (100 μm × 30 mm) filled with 3.5 μm X-Bridge BEH 130 C18 sorbent (Waters). After washing of trapping column with 0.1% FA, the peptides were eluted (flow rate 300 nl/min) from the trapping column onto an analytical column (Acclaim Pepmap100 C18, 3 µm particles, 75 μm × 500 mm; column compartment temperature of 40°C, Thermo Fisher Scientific) by 100 min nonlinear gradient program (0 min: 1%, 70 min: 30%, 100 min: 56% of mobile phase B; mobile phase A: 0.1% FA in water; mobile phase B: 0.1% FA in 80% ACN). Equilibration of the trapping column and the analytical column was done prior to sample injection to sample loop. The analytical column outlet was directly connected to the Digital PicoView 550 (New Objective) ion source with sheath gas option and SilicaTip emitter (New Objective; FS360-20-15-N-20-C12) utilization. ABIRD (Active Background Ion Reduction Device, ESI Source Solutions) was installed. MS data were acquired in a data-dependent strategy with defined number of scans based on precursor abundance with survey scan (350‒2000 *m*/*z*). The resolution of the survey scan was 60 000 (at *m*/*z* 400) with a target value of 1 × 10^6^ ions and maximum injection time of 1000 ms. HCD MS/MS (isolation window 1.4 *m*/*z*, 38% relative fragmentation energy) spectra were acquired with a target value of 5.0 × 10^4^, the maximum injection time 500 ms, and resolution of 15 000 (at *m*/*z* 400). Dynamic precursors exclusion was enabled for 45 s after one MS/MS spectra acquisition and the and total cycle time length was set to measure top10 precursor fragment spectra. The analysis of the mass spectrometric RAW data files was carried out using the MaxQuant software (version 1.6.2.10) using default settings unless otherwise noted. MS/MS ion searches were done against modified cRAP database (based on http://www.thegpm.org/crap) containing protein contaminants like keratin, trypsin etc., and UniProtKB protein database for *Homo sapiens* (https://ftp.uniprot.org/pub/databases/uniprot/current_release/knowledgebase/reference_proteomes/Eukaryota/UP000005640/UP000005640_9606.fasta.gz; version from 2018-05-28, number of protein sequences: 20996). Oxidation of methionine, deamidation (N, Q) and acetylation (protein N-terminus) as optional modification, carbamidomethylation (C) as fixed modification, and trypsin/P enzyme with two allowed missed cleavages were set. Peptides and proteins with FDR threshold < 0.01 and proteins having at least one unique or razor peptide were considered only. Match between runs was set among all analyzed samples. Protein abundance was assessed using protein intensities calculated by MaxQuant. Protein intensities reported in proteinGroups.txt file (output of MaxQuant) were further processed using the software container environment (https://github.com/OmicsWorkflows/KNIME_docker_vnc), version 4.1.3a. Processing workflow is available upon request. Briefly, it covered: (i) removal of decoy hits and contaminant protein groups, (ii) protein group intensities log2 transformation, (iii) LoessF normalization, (iv) proteins not quantified in at least two replicates of at least one sample type were filtered out (with at least two peptides in at least one replicate of single sample type), (v) missing values imputation per replicate using the imp4p package, and (vi) normalized and imputed protein intensities utilization for differential expression analysis using LIMMA statistical test with block factor specifying the samples preparation per replicate. Proteins with adjusted *P*-value < 0.05 and fold change > 2 were considered as potential interacting partners for a given comparison of IP samples with corresponding control. Experiment was done in three biological replicates.

### Immunofluorescence

HCT116 cells were grown on 12 mm cover slips for 2 days and subsequently inhibited with 100 nM SY-351 for 4 h. Control cells were incubated with DMSO. Cells were washed twice with PBS and fixed with 3.7% paraformaldehyde in PBS for 10 min. After fixation slides were washed twice with PBS, permeabilized with 0.5% Triton X-100 in PBS for 20 min, and washed 3 × 5 min in PBS. Blocking was done with 3% fetal bovine serum (FBS) in PBS for 1 h. Subsequently, cells were incubated with primary antibodies (P-CDK11 Ab1, P-CDK11 Ab2, 1:100 dilution), SF3B1 (MBL, MB-D221-3, 1:300 dilution), CDK11 (Abcam, ab19393, 1:400 dilution), and CDK7 (Bethyl, A300-405A, 1:400 dilution) for 1 h and washed 3 × 5 min with PBS. Cells were incubated with secondary antibodies (AlexaFluor 568 (Thermo Fisher, A-11011), Alexa Fluor 488 (Thermo Fisher, A-11017), and 1:500 dilution) for 1 h followed by washing 3 × 5 min with PBS. Finally, coverslips with cells were mounted on microscope slides using ProLong^TM^ Gold Antifade Mountant with DNA Stain DAPI (ThermoFisher, P36941). Images were obtained with an inverted microscope Zeiss Axio Observer.Z1/7 with confocal unit LSM 800 and Plan-APOCHROMAT ×63/1.4 oil objective, and Zen 2 software. To quantify the differences in signal intensities, cells from macrographs of two conditions were selected [DMSO (*n* = 121), SY-351 (*n* = 132)]. Using IMARIS 10 software, the pixel intensities of fluorescence signals within the defined region (nucleus) were measured. Results are expressed as median and interquartile range (IQR) of relative fluorescence intensity.

### Chromatin fractionation

Two 15-cm dishes of HCT116 cells at 70% confluency were inhibited with 50 nM SY-351 for 2 h. Another two dishes were treated with DMSO as a control. Cells were harvested, centrifuged 420 × *g*/4°C/5 min, pellets were resuspended in 4 ml of HLB + N buffer [10 mM Tris buffer, pH 7.5, 10 mM NaCl, 2.5 mM MgCl_2_, 0.5% NP-40, and Protease inhibitor cocktail (P8340, Sigma)] and incubated 5 min on ice. Swollen cells were underlaid with 1 ml of ice-cold HLB + NS buffer [10 mM Tris buffer, pH 7.5, 10 mM NaCl, 2.5 mM MgCl_2_, 0.5% NP-40, 10% sucrose, and Protease inhibitor cocktail (P8340, Sigma)], centrifuged 420 × *g*/4°C/5 min, 100 μl of supernatant was collected as cytoplasmic fraction, and the rest was discarded. Pellet was completely resuspended in 125 μl of NUN-1 buffer [20 mM Tris buffer, pH 7.9, 75 mM NaCl, 0.5 mM EDTA, 50% glycerol, Protease inhibitor cocktail (P8340, Sigma), and Phosphatase Inhibitor Cocktail 3 (Sigma, P0044)]. 1.2 ml of NUN-2 buffer [20 mM HEPES-KOH, pH 7.6, 300 mM NaCl, 0.2 mM EDTA, 7.5 mM MgCl_2_, 1% NP-40, 1 M urea, Protease inhibitor cocktail (P8340, Sigma), and Phosphatase Inhibitor Cocktail 3 (Sigma, P0044)] was added and samples were vortexed at maximum speed. Samples were incubated on ice for 16 min with vortexing every 3‒4 min and centrifuged 1000 × *g*/4°C/10 min, 100 μl of supernatant was collected as nucleoplasmic fraction, and 500 μl was used for glycerol gradient experiment. Pellets were washed 2 × with 1 ml of ice-cold NET-2 buffer (50 mM Tris buffer, pH 7.5, 150 mM NaCl, and 0.05% NP-40) + Protease Inhibitor Cocktail, Sigma, P8340) + Phosphatase Inhibitor Cocktail 3 (Sigma, P0044). Pellet was washed 1× with 100 μl of MNase mix [1 × MNase buffer (NEB, B0247S), 1 × BSA (NEB, M0247S)] and then incubated for 6 min with 100 μl of MNase [40 unit/μl MNase (NEB, B0247S), 1 × MNase buffer (NEB, B0247S), 1 × BSA (NEB, M0247S)] at 37°C, 1400 rpm shaking. Reaction was stopped by incubation and vortexing with 10 μl of 250 mM ethylene glycol-bis(2-aminoethylether)-N,N,N,N-tetraacetic acid (EGTA) (Sigma, E3889) at room temperature followed by addition of 450 μl of NET-2 buffer + Protease Inhibitor Cocktail, Sigma, P8340) + Phosphatase Inhibitor Cocktail 3 (Sigma, P0044). Samples were centrifuged 16 000 × *g*/4°C/5 min, 50 μl of supernatant was collected as chromatin fraction and 500 μl was used for glycerol gradient experiment or IP. Cytoplasmic, nucleoplasmic and chromatin fractions were mixed with 3 × Laemmli buffer and heated at 95°C for 3 min. Samples were resolved on western blot. Samples for glycerol gradient experiment or IP were kept on ice until experiment was performed on the same day.

### Immunoprecipitation of SF3B1 from a chromatin fraction

Samples were prepared as described above with the exception that fifteen 15-cm dishes of HCT116 cells at 70% confluence were used. Dynabeads G (25 μl/IP) were 3× washed in NET-2 buffer and incubated 3 h rotating at 4°C with 3 μg of SF3B1 antibody (MBL, MB-D221-3) or control mouse IgG (Proteintech, 66360-3-Ig). Beads were 3× washed in NET-2 buffer and chromatin sample was distributed between washed beads and incubated 90 min rotating at 4°C. Beads were 4× washed in NET-2 buffer + Protease Inhibitor Cocktail (Sigma, P8340) + Phosphatase Inhibitor Cocktail 3 (Sigma, P0044), mixed with 3× Laemmli buffer, and heated at 95°C for 3 min. Experiment was run in three biological replicates.

### Glycerol gradient

Glycerol gradients (10%‒40%, v/v) were prepared in NET-2 buffer on a Gradient Master Model 108 (Biocomp, Canada) and stored at 4°C until use. Gradients overlayed with 0.5 ml of sample were centrifuged for 19 h at 38 000 rpm in Sw41Ti rotor on a Beckman Coulter Optima XPN-80 ultracentrifuge. Fractions of 1 ml were collected manually, concentrated by trichloroacetic acid (TCA)/acetone precipitation, dissolved in 30 μl of 3× Laemmli buffer and heated at 95°C for 3 min. Experiment was run in two biological replicates.

### ChIP-Seq

ChIP was performed using the following antibodies for each technical replicate: 30 μl of P-CDK11 (Ab2 or Ab3), 5 μg of SF3B1 (MBL, MB-D221-3), and 30 μl of P-SF3B1 (T211 or T235). Two technical replicates were pooled together to obtain one biological replicate. In brief, 20 µl of Dynabeads protein G (Thermo Fisher Scientific, 10009D) per immunoprecipitation were pre-blocked with 0.2 mg ml^−1^ BSA (Thermo Fisher Scientific, AM2616) for 4 h, washed 3× with RIPA buffer, followed by incubation with the specific antibody at 4°C for at least 4 h. Where necessary, HCT116 cells (70% confluency) were treated with 50 nM SY-351 for 2 h. Cells were cross-linked with 1% formaldehyde for 10 min, and the reaction was quenched with glycine (final concentration 125 mM) for 5 min. Cells were washed 2× with ice-cold PBS, scraped, and split to several tubes so that there was about 20 µl packed cell pellet per tube. Each 20 µl packed cell pellet was lysed in 650 µl of RIPA buffer and sonicated 20 × 7 s (amplitude 0.85) using a 5/64 probe (QSonica Q55A). Supernatant (13 000 × *g*/4°C/10 min) was pooled and pre-cleared by incubating with Dynabeads protein G (Thermo Fisher Scientific, 10009D) at 4°C for 2‒4 h. Pre-cleared extracts were split into two technical replicates (900 µl each) and incubated overnight with antibody (P-CDK11, SF3B1, and P-SF3B1)-bound Dynabeads protein G. Beads were sequentially washed once with low-salt buffer [20 mM Tris–Cl, (pH 8), 150 mM NaCl, 2 mM EDTA, 1% Triton X-100, and 0.1% SDS], once with high-salt buffer (20 mM Tris–Cl, (pH 8), 500 mM NaCl, 2 mM EDTA, 1% Triton X-100, and 0.1% SDS), once with LiCl buffer [20 mM Tris–Cl, (pH 8), 250 mM LiCl, 2 mM EDTA, 1% NP-40, and 1% sodium deoxycholate] and 2 × with TE buffer (10 mM Tris–Cl, pH 8, and 1 mM EDTA). Immunoprecipitated complexes were eluted 2 × 15 min with 250 µl of elution buffer (1% SDS and 0.1 M NaHCO_3_). NaCl was added to final concentration of 0.2 M and samples were incubated at 65°C for at least 4 h to reverse cross-linking. Tris, pH 7.8, proteinase K (Sigma–Aldrich, P5568) and GlycoBlue (Thermo Fisher Scientific, AM9516) were added to final concentration of 30 mM, 0.04 mg/ml, and 0.06 mg/ml, respectively, and incubated at 42°C for 2 h. After phenol–chloroform extraction (Sigma–Aldrich, P3803), immunoprecipitated DNA was dissolved in 17 µl of water and technical replicates were pooled. ChIP-seq libraries were generated using KAPA Biosystems Hyper Prep Kit (Roche, KK8502) and NEBNext Multiplex Oligos for Illumina (New England Biolabs, E7335S, E7500S). Libraries were sequenced (50 bp single-end reads) using Illumina HiSeq 2500 system (VBCF, Vienna). Experiment was performed in two biological replicates.

### Purification of GST-tagged SF3B1 (1–463) fragment

GST-tagged SF3B1 (1–463) was purified as previously described [32].

### *In vitro* kinase assays

HCT116 cells were co-transfected with plasmid containing flag-tagged wild-type or mutant (T595A, T595D, T595E, S589A, S589D, and S589E) CDK11 together with plasmid containing CCNL1. Cells were harvested 48 h later, washed 2× with PBS, and lysed in 900 μl of IVKA lysis buffer [20 mM HEPES-KOH, pH 7.9, 300 mM KCl, 15% glycerol, 0.2% NP-40, and Protease Inhibitor Cocktail, Sigma, P8340)] for 15 min. Samples were sonicated on QSonica Q55A (5/64 probe) using 5 × 0.25 s pulse at 0.3 amplitude, centrifuged 10 000 × *g*/4°C/10 min and supernatant was transferred into new tube. Each sample was incubated for 1.5 h with 20 μl of pre-washed Flag agarose M2 affinity gel (Sigma, A2220) to immunoprecipitate Flag-CDK11. Samples were washed 3 × with High-salt buffer [20 mM HEPES-KOH, pH 7.9, 500 mM KCl, 15% glycerol, 0.2% NP-40, protease inhibitor cocktail (Sigma–Aldrich, P8340)], once with Detergent-free buffer (20 mM HEPES-KOH, pH 7.9, 150 mM KCl, and 15% glycerol), and kinases were eluted by 10-min incubation in Elution buffer [20 mM HEPES-KOH, pH 7.9, 150 mM KCl, 0.14 mg/ml 3 × Flag peptide (Sigma–Aldrich, F4799)]. Eluted kinases were mixed with truncated recombinant SF3B1 (aa 1–463) (purified as described above) in Kinase buffer (20 mM HEPES-KOH, pH 7.9, 5 mM MgCl_2_, and 1 mM ATP) and samples were incubated 30 min at 30°C, 300 rpm. Reactions were stopped by addition of 30 μl of 3 × Laemmli buffer and heating at 95°C for 3 min. Experiment was run in two biological replicates.

### Immunoprecipitation of CDK11 T-loop mutants

HCT116 cells with stably integrated FRT/TO plasmids containing flag-tagged wild-type or mutant CDK11 (T595A, T595D, and T595E) were induced by 1 μg/ml of doxycycline. In another set of experiments, HCT116 cells were co-transfected with plasmid containing flag-tagged wild-type or mutant (S589A, S589D, and S589E) CDK11. Cells were harvested 48 h later, washed 2× with PBS, and lysed in 1 ml of IVKA lysis buffer + Phosphatase Inhibitor Cocktail 3 (Sigma, P0044) for 10 min. Samples were sonicated on QSonica Q55A (5/64 probe) using 3 × 5 s pulse at 0.3 amplitude, centrifuged 10 000 × *g*/4°C/10 min, and supernatant was transferred into new tube. Each sample was incubated for 1.5 h with 15 μl of pre-washed Flag agarose M2 affinity gel (Sigma, A2220) to immunoprecipitate Flag-CDK11. Samples were washed 3× with High-salt buffer + Phosphatase Inhibitor Cocktail 3 (Sigma, P0044), mixed with 60 μl of 3 × Laemmli buffer, and heated at 95°C for 3 min. Experiment was run in two biological replicates.

### Preparation of lysates for western blotting analysis upon time-course inhibition of CDK7 and CDK11

HCT116 cells were treated with 50 nM SY-351 or 50 nM OTS964 for indicated times. Cells were harvested, 3× washed in ice-cold PBS, and lysed in 50 μl of RIPA buffer supplemented with Phosphatase Inhibitor Cocktail 3 (Sigma–Aldrich, P0044) for 10 min. Samples were sonicated on QSonica Q55A (5/64 probe) using 5 × 0.25 s pulse at 0.3 amplitude, centrifuged 10 000 × *g*/4°C/10 min, and protein concentration in supernatant was equalized using Pierce BCA Protein Assay Kit (Thermo Fisher, 23225). Samples were mixed with 3× Laemmli buffer and heated 95°C/3 min. Experiment was run in three biological replicates.

### qPCR

HCT116 cells were treated with 50 nM SY-351 or 50 nM OTS964 for 1, 2, 4, 6, or 8 h. RNA was extracted using Tri-Reagent (93289, Sigma–Aldrich). After DNase I treatment (AMPD1, Sigma–Aldrich), 1 μg of RNA was reverse-transcribed using random hexamers and Superscript II reverse transcriptase (Thermo Fisher Scientific, 18064014). The resulting complementary DNA (cDNA) was 40× diluted with water and 5 µl of diluted cDNA was used as a template for each quantitative polymerase chain reaction (qPCR) using SYBR Green JumpStart TaqReadyMix (Sigma–Aldrich, S4438) with the following parameters: 95°C for 2 min followed by 45 cycles of denaturation at 95°C for 15 s, annealing at 55°C for 30 s, and extension at 72°C for 30 s. Primers used in this study are listed in Supplementary Table S4. RT-qPCR assays were performed on the Aria Mx instrument (Agilent) in technical triplicate for each biological replicate and error bars represent SD of three biological replicates. *PPIA* gene was used as a housekeeping gene and DMSO-treated control as normalizer to calculate 2^−ΔΔCq^ [54]. Ratio of unspliced to spliced transcripts was calculated by dividing value of exon-intron primer pair by value of exon-exon primer pair. Experiment was run in three biological replicates.

### RT-PCR

HCT116 cells were treated with DMSO or 100 nM SY-351 for 0.5, 1, 2, 4, or 6 h. Cells were washed twice with ice-cold PBS, scraped, pelleted at 200 × *g*/3 min, and treated for 5 min with 150 µl of cytoplasmic lysis buffer (10 mM Tris buffer, pH 8, 0.32 M sucrose, 3 mM CaCl_2_, 2 mM MgCl_2_, 0.1 mM EDTA, 0.5% Triton X-100, and supplemented with 40 U ml^−1^ RNase inhibitor, Roche, 3335402001). Cytoplasmic RNA present in the supernatant was completely removed after centrifugation (500 × *g*/3 min). Nuclear RNA was extracted using Tri-Reagent (93289, Sigma–Aldrich). After DNase treatment (AMPD1, Sigma–Aldrich), 1 μg of RNA was reverse-transcribed using random hexamers and Superscript II reverse transcriptase (Thermo Fisher Scientific). PCR was performed in a 20 µl of reaction mixture containing 5 ng of the obtained cDNA (or 50 ng of human genomic DNA as a control), 1 × Phusion HF buffer, 0.2 mM dNTP mix, 0.5 µM of each primer, and 0.4 U Phusion DNA polymerase (F530S, Thermo Fisher Scientific). The PCR conditions were as follows: 94°C for 30 s, 35 cycles of 94°C for 10 s, 62°C for 20 s, and 72°C for 30 s, followed by 72°C for 10 min. PCR products were separated on a 1.5% agarose gel and stained with Midori Green (MG03, Nippon Genetics) and quantified using ImageJ (v.1.53f51). Experiment was run in two biological replicates.

### SILAC phosphoproteomics

Samples were prepared similar to [55]. SILAC DMEM Kit, U-13C, U-15N (Lys & Arg) (Silantes, 282986 444) was used to prepare “light” and “heavy” samples (gift of Dr. Falk Butter). Briefly, DMEM medium without glutamine, lysine, and arginine was supplemented with 0.798 mM L-arginine and 0.398 mM L-lysine, 10% dialyzed FBS, 2 mM L-glutamine (Silantes, 282986444), and 200 μg/ml of L-proline (Sigma, P5607). In “heavy” medium, ^13^C- and ^15^N-labeled variants of both L-lysine and L-arginine were used. HCT116 cells were grown in light or heavy media for 5‒10 cell doublings before the experiment. On the day of experiment, two 15-cm dishes (80% cell confluency) were treated with 50 nM OTS964 or DMSO for 10 min. In total, four biological replicates were performed—in two biological replicates, cells grown in light medium were inhibited and cells grown in heavy medium were treated with DMSO. In remaining replicates treatment was flipped and cells in heavy medium were inhibited instead. All lysis buffers were supplemented with Protease Inhibitor Cocktail (Sigma, P8340) and phosphatase inhibitors (50 mM β-glycerophosphate, 50 mM sodium fluoride, and 10 mM sodium orthovanadate). After 10-min inhibition, cells were washed twice with ice cold PBS, scraped to 1.5-ml tubes, and spun down. Cells were then resuspended in 1 ml of Cytoplasmic buffer (10 mM HEPES–KOH, pH 7.9, 10 mM KCl, 1.5 mM MgCl_2_, and 0.05% NP-40) by pipetting up and down and incubated on ice for 15 min. Nuclei were separated in centrifuge at 500 × *g*/5 min/4°C and washed again in 1 ml of Cytoplasmic buffer. Nuclei were lysed in 1 ml of RIPA lysis buffer (50 mM Tris–HCl, pH 7.5, 150 mM NaCl, 1 mM EDTA, 1% IGEPAL CA-630, and 0.1% sodium deoxycholate) for 15 min on ice, before addition of 1/10 volume (120 μl) of 5 M NaCl. Lysates were mixed and sonicated on QSonica Q55A (5/64 probe) using 4‒6 × 5 s pulse at 0.3 amplitude. Lysates were centrifuged at 16 000 × *g*/4°C/10 min, and protein concentration in supernatant was measured using BCA assay kit (Pierce, 23225). 5 mg of control sample was mixed with equal amount of inhibited sample, and proteins were precipitated by addition of 4 volumes of ice-cold acetone at −20°C overnight. Next day, acetone-precipitated proteins were pelleted by centrifugation at 2500 × *g*/4°C/10 min. Supernatant was discarded and protein pellet was resuspended in 4 ml of Denaturation buffer (10 mM HEPES–NaOH, pH 8, 8 M urea). Disulfide bonds were reduced by 1 mM DTT for 1 h at room temperature followed by alkylation using 5.5 mM 2-chloroacetamide (Sigma, C0267) for 1 h at room temperature. Proteins were then digested by addition of 100 μg of LysC (Wako) for 6 h at 37°C before diluting the sample with 4× volume of 50 mM ammonium bicarbonate (Sigma, A6141) and addition of 200 μg of trypsin (Sigma, T6567) and digesting overnight at 37°C. Next day, 5 μl of sample was mixed with 5 μl of 5% FA for proteome analysis, and remaining sample was acidified by addition of trifluoroacetic acid (TFA, Sigma, T6508) to a final concentration of 0.5%. Samples were incubated for 1 h at 4°C, then centrifuged 4000 × *g*/4°C/10 min. Supernatants containing tryptic peptides were desalted on Sep-Pak C18 Classic Cartridge (Waters, WAT0519510). Briefly, C18 columns were washed once with 5 ml of 100% ACN, followed by three washes with 5 ml of 0.1% TFA in water. Peptide solution was loaded onto the C18 columns and columns were washed three times with 5 ml of 0.1% TFA in water. Peptides were eluted with 4.5 ml of 50% ACN and 0.1% TFA in water.

Peptide concentration in the eluate was estimated using total ion chromatogram of LC-MS analysis using RSLCnano system (Thermo Fisher Scientific) online connected to Impact II mass spectrometer (Bruker) using external calibration done using HeLa digest peptide mixture (Pierce, 88328). The eluted desalted peptides were split into three aliquots in the following way: 1/100 was set apart for global proteome analysis and the remaining part was split into two equal parts for phosphopeptide enrichment. All aliquots were dried using SpeedVac (Thermo Fisher Scientific) and stored at −80°C upon further processing.

Aliquots for global proteome analyses (herein labeled as “ID” solutions) were transferred using 2.5% FA in 50% ACN and 100% ACN into the LC-MS vial and ACN was removed by vacuum (SpeedVac, Thermo Fisher Scientific) prior LC-MS analyses. Aliquots for the phosphopeptide enrichment were processed in parallel using High-Select^™^ TiO2 Phosphopeptide Enrichment Kit (Thermo Fisher Scientific, A32993) or High-Select^™^ Fe-NTA phosphopeptide Enrichment Kit (Thermo Scientifics, A32992), according to manufacturer’s protocol. Each aliquot was divided in half and each half was processed separately by each enrichment type and pooled back prior further processing. Pooled phosphopeptide aliquots after enrichments were evaporated in a SpeedVac and stored at −80°C before further processing. Phosphopeptides were solubilized in 150 μl of 0.1% TFA in Milli-Q water and 15 μl were taken for pre-fractionation analysis; the rest were used for fractionation using a home-made stage-tips for strong cation exchange chromatography (SCX tips). 17-gauge Hamilton syringe was used for cutting 6 disks from 47 mm Empore^™^ SPE disk (66886-U, Sigma–Aldrich) and transferring them into 200 μl tip. SCX wash buffer (40% ACN and 0.1% TFA) and SCX elution buffers (40 mM acetic acid, 40 mM boric acid, and 40 mM phosphoric acid; the pH was adjusted with sodium hydroxide to 3.0, 3.5, 4.0, 4.5, 6.5, and 8.5) were prepared fresh. Due to nature of home-made tips, adjustment of centrifugation time for each washing step is needed to avoid drying SCX tips. SCX tips were washed with 50 μl of methanol and centrifuged for 30 s at 500 *× g*. Then, SCX tips were washed with 50 μl of SCX elution buffer, pH 8.5, centrifuged for 50 s at 500 × g and with 50 μl of SCX washing buffer, and centrifuged for 50 s at 500 × *g*. After that, phosphopeptide samples were loaded on SCX tips and centrifugated for 2 min at 400 × *g*. Peptides were fractioned with SCX elution buffer, 100 μl of SCX elution buffer, starting from pH 3.0, was pipetted on SCX tip and centrifuged for 2 min at 400 × *g*; the elution steps were repeated with another SCX elution buffers with increasing pH, ending with pH 8.5. Each eluate was collected into new 2 ml of tube. In the end, six fractions were collected per starting sample. All fractions were concentrated in a SpeedVac to volume 15‒40 μl. After that, pH of all fractions was adjusted to pH 2‒3 using 0.1%, 1%, and 10% TFA. All fractions were desalted using home-made C18 stage tips (C18 tips). For stage tip preparation, 17-gauge Hamilton syringe was used to cut 2 disks from 47 mm Empore^™^ C18 disk (Agilent, USA) into a 200 μl of tip. Buffer A (0.1% FA), buffer B (80% ACN and 0.1% FA), and elution buffer (50% ACN and 0.1% FA) were prepared fresh. Due to nature of home-made tips, adjustment of centrifugation time for each washing step is needed to avoid drying C18 tips. C18 tips were washed with 25 μl of methanol and centrifuged for 20 s at 500 × *g*. Before methanol left the C18 tips, 25 μl of buffer B was added and centrifuged for 40 s at 500 × *g*. After that, C18 tips were washed twice with 25 μl of buffer A and centrifuged for 1 min at 500 × *g*. The C18 tips were transferred into new 2 ml of tubes. Each fraction was loaded and centrifuged for 5 min at 400 × *g*; the flow-through was loaded again and centrifuged for 2 min at 400 × *g*. C18 tips were washed with 50 μl of buffer A, which was used to rinse the tubes, where the fractions were, and centrifuged for 5 min at 400 × *g*. Peptides were eluted directly into LC-MS vials with already added PEG (final concentration 0.001%) by passing 50 μl of elution buffer through the C18 tips using a syringe. Phosphopeptide fractions were concentrated in a SpeedVac under 15 μl, 1.5 μl of 5% FA was added and the volume was adjusted by Milli-Q water to 15 μl. Phosphopeptide fractions were analyzed by LC-MS/MS. Phosphopeptide fractions are herein labeled as “Ti” and “IM” solutions based on applied phosphoenrichment type (TiO_2_ and IMAC), with additional “FX” suffix where the “X” represents the fraction number.

LC-MS/MS analyses of all peptide samples were done using UltiMate 3000 RSLCnano system (Thermo Fisher Scientific) connected to Orbitrap Exploris 480 mass spectrometer (Thermo Fisher Scientific). Prior to LC separation, tryptic digests were online concentrated and desalted using trapping column (Acclaim PepMap 100 C18 Neo, dimensions 300 μm ID, 5 mm long, 5 μm particles, Thermo Fisher Scientific; 174500). After washing of trapping column with 0.1% TFA, peptides were eluted in backflush mode (flow rate 250 nl/min) from the trapping column onto an analytical separation column (EASY-Spray column, 75 μm ID, 500 mm long, 2 μm particles, Thermo Fisher Scientific; ES903) by 120 min gradient program (3%‒38% of mobile phase B; mobile phase A: 0.1% FA in water; mobile phase B: 0.1% FA in 80% ACN). Trap column was operated at ambient temperature and analytical column at 50°C.

MS data were acquired in a data-dependent strategy (cycle time 3 s). Survey scan range was set to *m/z* 350‒2000 with the resolution of 120 000 (at *m/z* 200), normalized target value of 250%, and maximum injection time of 500 ms. HCD MS/MS spectra (isolation window *m/z* 1.2, 30% relative fragmentation energy) were acquired from 120 *m/z* with the default AGC target, resolution of 30 000 (at *m/z* 200) and maximum injection time set to Auto. Dynamic exclusion was enabled for 45 s. Targeted Mass Difference node was added to the method workflow with number of precursors in the target group set to 2 (Lys8 delta mass of 8.0142 and Arg10 delta mass of 10.0083), intensity range of 10%‒100%, ±5 ppm tolerance and selecting the most intense precursor requiring the same precursor charge state. Samples for the SILAC labels incorporation check were measured using slightly differing MS method (DDA, without Targeted Mass Filter node, MS/MS spectra AGC target value of 50% and 50 ms maximum injection time).

All acquired raw LC-MS data for all three solutions (ID, IM, and Ti) and all SCX fractions measured for each sample were processed together using MaxQuant software (v2.0.3.0) [56] with inbuild Andromeda search engine [57]. Fractions design was set to enable match between runs among all replicates of individual solutions type (ID, IM, and Ti) but not between them. Phosphopeptide enriched solutions (IM and Ti) had set PTM flag to True and were set to a separate parameter group with phospho (STY) variable modification set on top of the other settings common with ID solution analyses. The experiment type was set to Standard with multiplicity level of 2 (Light, no modification; Heavy, Arg10, Lys8). The search was done against protein databases of Homo sapiens (20593 protein sequences, version from 2023-05-03, downloaded from https://ftp.uniprot.org/pub/databases/uniprot/current_release/knowledgebase/reference_proteomes/Eukaryota/UP000005640/UP000005640_9606.fasta.gz) and cRAP contaminants (112 sequences, version from 2018-11-22, downloaded from http://www.thegpm.org/crap). Modifications were set as follows for database search: oxidation (M), deamidation (N, Q), and acetylation (Protein N-term) as variable modifications, with carbamidomethylation (C) as a fixed modification. Enzyme specificity was tryptic with two permissible miscleavages. Only peptides and proteins with false discovery rate threshold under 0.01 were considered. Re-quantify option as well as match between run features were enabled. SILAC labels incorporation data were searched using similar approach in MaxQuant, specifying the SILAC labels as variable modifications only. To evaluate global proteome changes, the MaxQuant txt report files were further processed using the software container environment (https://github.com/OmicsWorkflows/KNIME_docker_vnc), version 4.1.3a. Processing workflow is available upon request. Protein level information processing steps were as follows: (i) reverse hits and contaminant protein groups (cRAP) removal; (ii) individual channels protein intensities calculation for individual raw files using total protein intensity per raw file (Intensity columns) and corresponding normalized H/L ratios (Ratio H/L columns); (iii) protein group intensities (step 2) log2 transformation, normalization (loessF); (iv) statistical evaluation of normalized log2 intensities (step 3) using limma R package considering the SILAC pairs design, the channels swap and with resulting *P*-values adjustment using Benjamini and Hochberg method.

Phosphopeptides were processed starting from evidence.txt and getting some additional annotation information from Phospho (STY) Sites.txt and proteinGroups.txt tables and it covered the following steps: (i) evidences list filtering for those with at least 1 phosphorylation; (ii) removal of peptides coming from contaminating (cRAP) proteins; (iii) remaining evidences grouping on the sequence and modifications concatenated information (sum of all rows in intensity columns and median from ratio columns were returned) with additional pivoting using the experiment column [TiO_2_ (Ti) and IMAC (IM) enriched results kept separately]; (iv) calculation of sum or median of intensities or not normalized *H/L* ratios, respectively, coming from IM and Ti solution analyses of the same sample replicate; (v) individual channels (*H* or *L*) intensities calculation using the summed intensity (IM and Ti raw files) and corresponding median *H/L* ratios from the previous step; (vi) log2 transformation and loessF normalization of channel intensities; (vii) statistical evaluation of differences between inhibitor and control sample replicates using log2 normalized intensities from the previous step using limma R package, considering the SILAC pairs design, the channels swap.

MaxQuant search results for SILAC incorporation test were processed using the software container environment (https://github.com/OmicsWorkflows/KNIME_docker_vnc), version 4.7.7a. Processing workflow is available upon request. Briefly, the workflow covered the following steps of modificationSpecificPeptides.txt file processing: (i) peptides containing exactly one K or R were selected for the SILAC labels incorporation check only; (ii) peptides harbouring any other modification than SILAC related ones were removed leaving unmodified or singly modified (either on K or R) peptides only; (iii) intensity ratio was calculated for the SILAC modified and unmodified peptide in this way (separately for the K and R containing peptides): modified peptide ratio = modified peptide intensity/(modified peptide intensity + unmodified peptide intensity) * 100; (iv) histogram of values from the previous step was plotted and modus was considered as the labeling efficiency (>99% in both K and R containing peptides).

### *In vitro* splicing assay

*In vitro* splicing assay was performed as previously described [32] using SY-351 inhibitor.

### Bioinformatics analysis

ChIP-seq reads were aligned to the human genome (hg38) using BWA [58]. Reads with an alignment score <20 were discarded and duplicates were removed with the samtools rmdup function [59]. All steps were implemented and run using the Watchdog workflow management system [60].

The metagene analysis of read coverage distribution in ChIP‐seq data was restricted to high confident transcripts of protein‐coding genes annotated in Ensembl version 100. Transcripts shorter than 3180 bp were excluded. Furthermore, only genes were included with a distance of ≥5000 nt from the next gene. For each gene, we selected the transcript with the most read counts in the RNAPII DMSO ChIP‐seq samples from our previous study (normalized to library size) in the ±3 kb regions around the transcription start site (TSS) and transcription termination site (TTS). For each gene, the regions −3 kb to +1.5 kb of the TSS and −1.5 kb to +20 kb of the TTS were divided into 50 and 100 bp bins, respectively, and the remainder of the gene body (+1.5 kb of TSS to −1.5 kb of TTS) into 180 bins of variable length in order to compare genes with different lengths. For each bin, the average coverage per genome position was then calculated and normalized to sequencing depth. Finally, metagene plots were created by averaging results for corresponding bins across all genes considered and then averaging across replicates. To determine statistical significance of differences between inhibitor and control, paired Wilcoxon signed rank tests were performed for each bin comparing normalized coverage values for each gene for this bin with and without the inhibitor. *P*‐values were adjusted for multiple testing with the Bonferroni method across all bins within each subfigure and are color‐coded in the bottom track of each subfigure: red = adj. *P*‐value ≤ 10^−15^; orange = adj. *P*‐value ≤ 10^−10^; yellow: adj. *P*‐value ≤ 10^−3^.

## Results

### CDK7 transiently associates with CDK11

**Figure 1.**
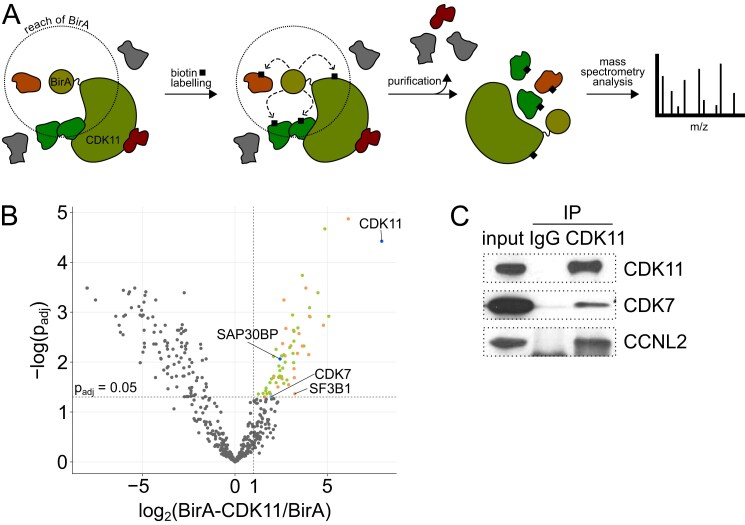
CDK7 transiently binds CDK11. (**A**) Schematic representation of proteomic BioID experiment for identification of proteins proximal to CDK11. The N-terminus of CDK11 fused to biotin ligase (BirA) biotinylates nearby proteins in cells. The biotinylated proteins are purified and identified by mass spectrometry. (**B**) Volcano plot of proteins identified in CDK11 BioID. Splicing factors are marked in orange, components of the CDK11 complex in blue, CDK7 in cyan and other proteins in green. (**C**) Immunoblot analysis of immunoprecipitations of endogenous CDK11 after 4 h treatment with 50 nM SY-351 in HCT116 cells. Detected proteins are indicated on the right. IgG = antibody control.

To determine neighboring and potentially interacting proteins of CDK11, we performed a proximity-dependent biotin identification (BioID) screen [52, 61] in HCT116 cells stably expressing control biotin ligase (BirA) or biotin ligase fused to CDK11’s N-terminus (BirA-CDK11) (Fig. 1A). To identify proteins proximal to CDK11, we combined the replicates and identified proteins enriched in BirA-CDK11 over control BirA (log_2_FC ≥ 1, *P*_adj _< 0.05) (Fig. 1B, and Supplementary Fig. S1A and Supplementary Table S1). In addition to CDK11, the CDK11 complex subunit SAP30BP [33], and various splicing factors, including the CDK11 substrate SF3B1 [32], we also identified CDK7 (Fig. 1B). We hypothesized that CDK7 could be a CDK11-activating kinase and its upstream regulator. Initially, we were unable to co-immunoprecipitate CDK7 with CDK11 in cells, which is not surprising, as the interaction between an enzyme and its substrates are usually transient. As the inhibition of an enzyme can “lock/freeze” its interaction with a substrate [62], we treated cells with CDK7 inhibitor SY-351 [11] and performed immunoprecipitation of CDK11. This experiment showed the association of CDK11 with CDK7 and its constitutive partner MAT1 [8], suggesting an existing, but transient interaction (Fig. 1C and Supplementary Fig. S1B).

### Characterization of CDK11 phosphorylated at canonical activating Thr595 in cells

**Figure 2.**
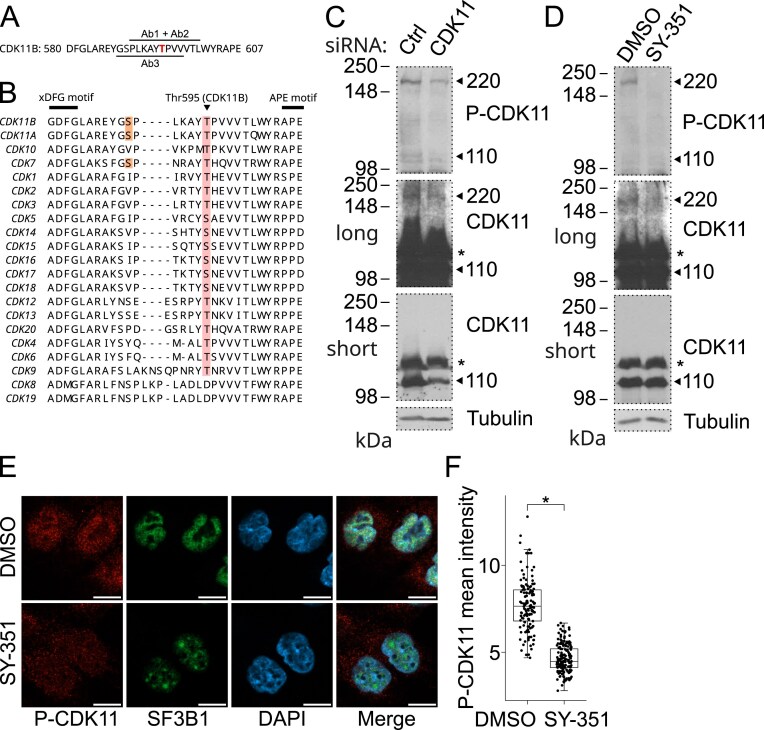
CDK11 is phosphorylated on canonical activating Thr595 in cells. (**A**) Amino acid sequence of T-loop of CDK11B. Activating Thr595 is in red. Peptide sequences used for the production of three (Ab1, Ab2, and Ab3) phospho-specific Thr595 antibodies are indicated by black bars. (**B**) Multiple protein sequence alignment of T-loop of CDKs. Activating Thr residues are highlighted in red, Ser corresponding to Ser164 in CDK7 [71] is in orange. xDFG and APE motifs are indicated. (**C**) Immunoblot analysis of CDK11 in HCT116 cells upon treatment with control or CDK11 siRNAs for 40 h. Bands corresponding to CDK11^110^ and P-CDK11^220^ (monitored by Ab3) are marked on the right with arrows. “Long” and “short” corresponds to long and short exposure of the film. Asterisk denotes nonspecific bands. (**D**) Immunoblot analysis of CDK11 in HCT116 cells upon treatment with 50 nM SY-351 or control DMSO for 4 h. See Fig. 2C for the legend. (**E**) Immunofluorescence microscopy of HCT116 cells using DAPI stain and the P-CDK11^220^ (Ab2) and SF3B1 antibodies upon 100 nM treatment with SY-351 or control DMSO for 4 h; scale bar = 10 µm. (**F**) Quantification of P-CDK11 intensity (monitored by Ab2) in Fig. 2E. Box plots represent median and IQR, whiskers extend to the furthest value inside 1.5 × IQR, asterisk: *P* < 0.05.

To investigate whether CDK7 is a CDK11-activating kinase, we made antibodies that recognize phosphorylated Thr595 in the activation T-loop of CDK11 (P-CDK11). We raised three different antibodies (Ab1, Ab2, and Ab3) recognizing two different phospho-specific CDK11 epitopes (Fig. 2A). The epitopes are unique for CDK11 and are not found in other CDKs (Fig. 2B) or other human proteins. Surprisingly, western blotting analyses of cell lysates revealed that all phospho-specific CDK11 antibodies recognize an ∼220 kDa band (Fig. 2C, and Supplementary Fig. S2A and B). The sensitivity of the bands to CDK11 siRNA (Supplementary Fig. S2A) and alkaline phosphatase treatments (Supplementary Fig. 2B) suggested identification of P-CDK11 as a 220 kDa protein. The expected 110 kDa bands of CDK11 were only detected with Ab2 and weakly with Ab3 (Fig. 2C, and Supplementary Fig. S2A and B). The 110 kDa bands decreased upon siRNA CDK11 depletion (Supplementary Fig. S2A), but not upon phosphatase treatment (Supplementary Fig. S2B), suggesting that the antibodies to some extent also recognize the nonphosphorylated 110 kDa form of CDK11 (Supplementary Fig. S2A and B). To further confirm that the identified 220 kDa band corresponds to CDK11, we probed western blots of cell lysates with the antibody raised against the N-terminus of CDK11 [45]. The 220 kDa band was only apparent after longer film exposure and was ~50–100 times weaker than the band corresponding to 110 kDa CDK11. Both bands were sensitive to the CDK11 siRNA treatment (Fig. 2C). Western blotting analyses of lysates from cells where the expression of stably integrated flag-CDK11 was induced by doxycycline [45] resulted in the detection of a 220 kDa band by flag antibody (Supplementary Fig. S2C). In summary, these experiments confirmed that the 220 kDa band corresponds to CDK11.

CDK11 was reported to form homodimers [63, 64]. By co-expression and co-immunoprecipitation of flag-tagged wild-type and deletion mutant of CDK11 proteins (Supplementary Fig. S2D) with myc-tagged wild-type CDK11, we confirmed that CDK11 can homodimerize in cells, and that different CDK11 domains contribute to the homodimerization (Supplementary Fig. S2E). Although it is generally accepted that the denaturing conditions of SDS–PAGE disrupt protein complexes, it was recently reported that some proteins with IDRs such as AKAP95 and HEXIM1 can form dimers strongly resistant to denaturing conditions [65, 66]. Thus, we conclude that the 220 kDa band corresponds to a subset of CDK11, likely to an SDS-resistant CDK11 dimer, and that this form of CDK11 is preferentially phosphorylated at the canonical activating Thr595 in the T-loop in cells. We denote this form of CDK11 as CDK11^220^ and 110 kDa CDK11 as CDK11^110^ in the text below.

In previous work, CDK7-catalyzed phosphorylation of CDK11 was detected in CDK11^110^, and using recombinant, mitosis-specific CDK11^58^ in reticulocyte lysate, the phosphorylation was mapped to the canonical activating Thr residue in the T-loop [67]. Since it was reported that CDK11^58^ is hard to detect in many cell lines [3, 33, 41, 42], we decided to determine the endogenous expression of CDK11^58^ in HCT116 cells. We performed western blotting of HCT116 control and siRNA-treated cell lysates, and probed them with antibodies directed against the C-terminal kinase domain of CDK11. While the band corresponding to CDK11^110^ diminished upon treatment with three different CDK11 siRNAs, we did not detect a siRNA-sensitive band of CDK11^58^ (Supplementary Fig. S2F). Likewise, we were unable to find any proof of the expression of CDK11^58^ in cells synchronized and released from a double thymidine block (Supplementary Fig. S2G). The proper synchronization of cells in G1/S was confirmed by the expression of G1-specific cyclin E2 (CCNE2) (Supplementary Fig. S2G). It is worth noting that a weak ∼58 kDa band observed in both experiments was neither sensitive to siRNA nor cell cycle phase, thus we consider it to be unspecific (Supplementary Fig. S2F and G).

In summary, we identified a subset of CDK11, herein termed CDK11^220^, that is phosphorylated in the T-loop in cells, and is likely a denaturing condition-resistant dimer of CDK11^110^. Moreover, we did not find any evidence of the expression of CDK11^58^ in HCT116 cells.

### CDK7 kinase activity is required for the formation of P-CDK11^220^ in cells

Having characterized P-CDK11 antibodies and various forms of CDK11 proteins in cells, we investigated the role of CDK7 in CDK11 T-loop phosphorylation. Treatment with SY-351 resulted in the complete loss of both P-CDK11^220^ and CDK11^220^ from cell lysates, while CDK11^110^ levels were not affected (Fig. 2D). This suggests that CDK7 inhibition results in the dephosphorylation of CDK11^220^ and a decrease in its levels in cells. CDK7-specific decrease of P-CDK11^220^ was confirmed by the inhibition of CDK7 with the competitive ATP analog 3MB-PP1 in cells carrying endogenous analog-sensitive (AS) alleles of CDK7 (CDK7^AS/AS^) [9] (Supplementary Fig. S2H). To determine the subcellular localization of P-CDK11, we performed immunofluorescence microscopy with the P-CDK11 antibodies. This experiment showed that P-CDK11 predominantly localized into nuclei, where it preferentially resided in nuclear speckles, as suggested by the overlap with the nuclear speckle marker SF3B1 [68] (Fig. 2E and Supplementary Fig. S2I). As expected, SY-351 treatment significantly decreased the P-CDK11 signal in the nuclei (Fig. 2E and F, and Supplementary Fig. S2I and J) and altered the association of SF3B1 with nuclear speckles, as reported previously [11]. A weak signal of P-CDK11 in cytoplasm was not affected by SY-351 treatment (Fig. 2E and Supplementary Fig. S2I) (see further results). Additionally, total CDK11 and CDK7 localised into nuclei, where CDK11 preferentially and CDK7 partially resided in nuclear speckles (Supplementary Fig. S2K). This is consistent with the localization of P-CDK11 and CDK7 acting on CDK11. We conclude that P-CDK11^220^ is dependent on CDK7 kinase activity.

### P-CDK11^220^ is associated with active spliceosome on chromatin

**Figure 3.**
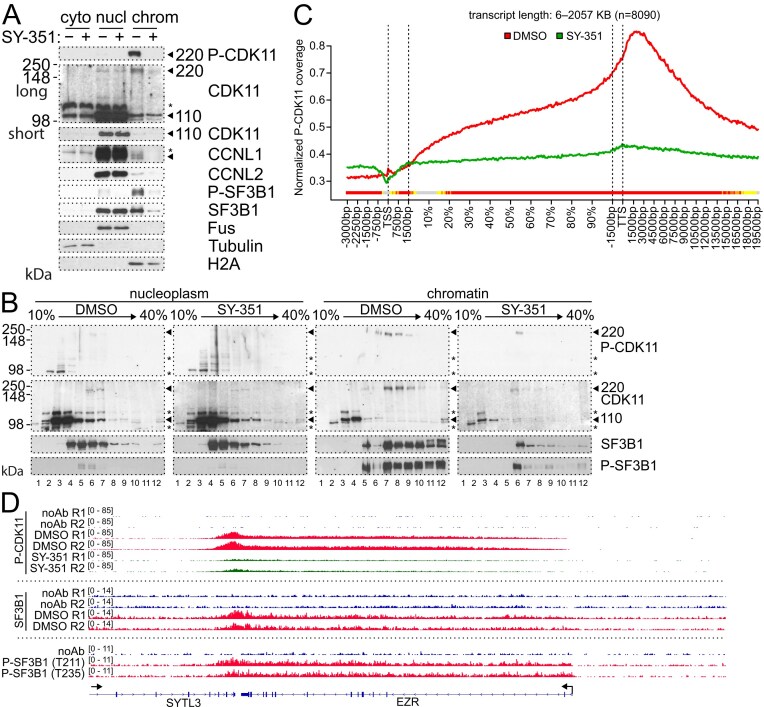
CDK7 is required for the formation of P-CDK11^220^ and active spliceosomes. (**A**) Immunoblot analyses of indicated proteins in HCT116 cells treated with control DMSO or 50 nM SY-351 for 2 h and separated into cytoplasmic (cyto), nucleoplasm (nucl), and chromatin (chrom) fractions. Bands corresponding to CDK11^110^ and P-CDK11^220^ (monitored by Ab3) are marked on the right with arrows. CCNL1 is marked on the right with an arrow. “Long” and “short” corresponds to long and short exposure of the film. Asterisk denotes a nonspecific band. (**B**) Immunoblot analyses of indicated proteins in nucleoplasm and chromatin fractions (from Fig. 3A) separated by ultracentrifugation in 10%–40% glycerol gradient. Bands corresponding to CDK11^110^ and P-CDK11^220^ (monitored by Ab3) are marked on the right with arrows. Asterisks denote nonspecific bands. (**C**) Metagene analyses of P-CDK11 ChIP-Seq (using Ab2) on 8090 genes in cells treated with either control DMSO or 50 nM SY-351 for 2 h. Each transcript was divided into two parts with fixed length [transcription start site (TSS) −3 kb to +1.5 kb and transcription termination site (TTS) −1.5 kb to +20 kb] and a central part with variable length corresponding to the rest of gene body (shown in %). Each part was binned into a fixed number of bins (90/180/215), average coverage normalized to sequencing depth was calculated for each bin for each transcript in each sample and then averaged first across genes and second across samples. The color track at the bottom indicates the significance of paired Wilcoxon tests comparing the normalized transcript coverages for each bin between DMSO and SY-351 treatment. *P*‐values are adjusted for multiple testing with the Bonferroni method within each subfigure; color code: red = adjusted *P*‐value ≤ 10^−15^, orange = adjusted *P*‐value ≤ 10^−10^, yellow = adjusted *P*‐value ≤ 10^−3^. (**D**) IGV genome browser view of P-CDK11^220^ ChIP-Seq on *EZR* gene in HCT116 cells treated with either control DMSO or 50 nM SY-351 for 2 h. SF3B1 and P-SF3B1 ChIP-seq on *EZR* gene in cells treated with control DMSO are shown below. noAb = control without antibody, R1, R2 = replicate 1, 2. T211 and T235 Ab = antibodies against P-Thr211 and P-Thr235 in SF3B1, respectively. *Y*-axis scale is denoted in square brackets.

To further determine the cellular localization of P-CDK11^220^, we performed subnuclear fractionation [69] in cells with or without 50 nM SY-351 treatment for 2 h. Optimal fractionation to cytoplasmic, nucleoplasmic and chromatin fractions were verified by the presence of tubulin, FUS, and histone 2a (H2A), respectively (Fig. 3A). P-CDK11^220^ was present almost exclusively in the chromatin fraction, together with small proportions (∼1–5%) of CDK11^110^, CCNL1, and CCNL2. The majority (∼90%–95%) of CDK11^110^, CCNL1, and CCNL2 resided in the nucleoplasm, and a small fraction (1%–5%) of CDK11^110^ and CCNL1 was in the cytoplasm (Fig. 3A). The SY-351 treatment led to a loss of P-CDK11^220^/CDK11^220^, CCNL1 and CCNL2 from the chromatin, while the levels of chromatin-bound CDK11^110^ remained approximately the same (Fig. 3A). This experiment shows that the presence of P-CDK11^220^ on the chromatin is dependent on CDK7, and that the release of CCNL1 and CCNL2 from the chromatin accompanies the reduction in the level of chromatin-bound P-CDK11^220^/CDK11^220^. Notably, CDK7 inhibition also resulted in a loss of P-SF3B1 from the chromatin (Fig. 3A). P-SF3B1 is a marker of the active spliceosome that mostly associates with the chromatin, while unphosphorylated SF3B1 is predominantly in the nucleoplasm [32, 35, 36, 70]. This can be visualized by western blotting analyses of cell lysates separated by glycerol gradient centrifugation, where large complexes of chromatin-associated active spliceosomes (marked by the presence of P-SF3B1) are separated from smaller complexes of mostly inactive spliceosome [35]. To determine the potential association of CDK11 with the active spliceosome, we carried out glycerol gradient sedimentation analyses of the chromatin and nucleoplasm fractions from the samples presented in Fig. 3A. Chromatin associated-P-CDK11^220^ exhibited faster sedimentation and co-fractionated with the active spliceosome marked by the presence of P-SF3B1 [35] (Fig. 3B, fractions 7–12). A small proportion (5%–10%) of CDK11^110^ was also bound to the chromatin, but in fractions not overlapping with the active spliceosome (Fig. 3B, fractions 2–4), suggesting that under these conditions the chromatin-bound CDK11^110^ mostly does not associate with the active spliceosome. The nucleoplasm contained most (∼90%–95%) of CDK11^110^ (Fig. 3B, fractions 2–6), and only unphosphorylated SF3B1 (Fig. 3B). The SY-351 treatment led to a loss of most of the active spliceosome, CDK11^220^, and some CDK11^110^ from the chromatin (Fig. 3B). To determine whether P-CDK11^220^/CDK11^220^ associates with the chromatin-associated spliceosome we immunoprecipitated a core spliceosome component SF3B1 from the chromatin cellular fraction. The observed co-immunoprecipitation of P-CDK11^220^/CDK11^220^ with SF3B1 supports its association with the spliceosome (Supplementary Fig. S3A). We were also able to co-immunoprecipitate CDK11^110^ (Supplementary Fig. S3A), suggesting that under these conditions this form of CDK11 also associates with chromatin-bound spliceosomes. Taken together, these experiments (Fig. 3A and B, and Supplementary Fig. S3A) suggested that CDK11^220^ is phosphorylated on its T-loop, bound to the chromatin, and associates with active spliceosome in cells. To further substantiate the presence of P-CDK11^220^ on the chromatin of genes, we performed ChIP-seq with two phospho-specific CDK11 antibodies (Fig. 2A, and Supplementary Fig. S2A and B). The metagene analyses of 8090 protein-coding genes showed broad P-CDK11^220^ occupancy over gene bodies, with some accumulation at and beyond transcription termination sites (Fig. 3C and Supplementary Fig. S3B). The SY-351 treatment resulted in a loss of P-CDK11^220^ chromatin binding, confirming the specificity of the ChIP-seq signal (Fig. 3C). Inspection of the individual genes confirmed the results of the metagene analyses (Fig. 3D and Supplementary Fig. S3C). A comparison of total SF3B1, P-SF3B1, and P-CDK11^220^ ChIP-seq occupancies on individual genes showed their mutual overlap over gene bodies (Fig. 3D and Supplementary Fig. S3C), supporting the association of P-CDK11^220^ with the chromatin-associated active spliceosome on gene bodies [36, 70]. We conclude that P-CDK11^220^ is present on the chromatin and associates with the active form of the spliceosome.

### Thr595 in the T-loop is essential for CDK11 kinase activity and CCNL/SAP30BP association

**Figure 4.**
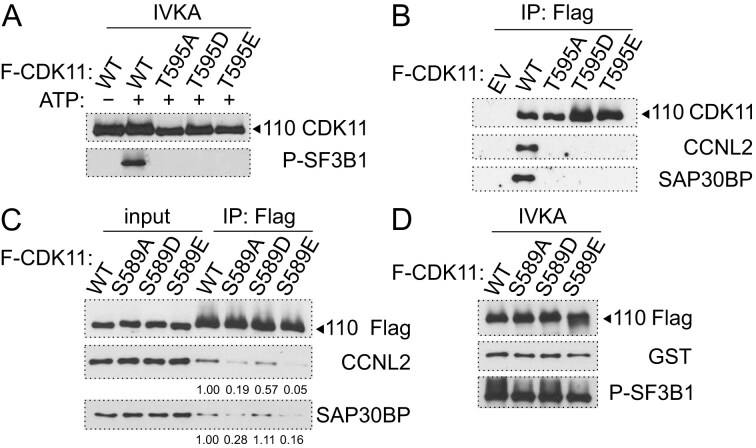
Thr595 in CDK11 is essential for the formation of CDK11/CCNL/SAP30BP complex. (**A**) Immunoblot of IVKAs with flag-tagged CDK11 wild-type (WT) and Thr595 mutants (T595A, T595D, and T595E) on GST-SF3B1 (1–463) substrate. The phosphorylation was monitored by the indicated antibody. Presence of ATP in IVKA is indicated. (**B**) Immunoblot analyses of immunoprecipitations of flag-tagged wild type (WT) or Thr595 mutants (T595A, T595D, and T595E) from HCT116 cells. Detected proteins are indicated on the right. EV = empty vector. (**C**) Immunoblot analyses of immunoprecipitations of flag-tagged WT or Ser589 mutants (S589A, S589D, and S589E) from HCT116 cells. Detected proteins are indicated on the right. Quantity of the immunoprecipitated proteins (CCNL1 and SAP30BP) is shown and was normalized to WT that equals 1. (**D**) Immunoblot of IVKAs with flag-tagged CDK11 WT and Ser589 mutants (S589A, S589D, and S589E) on GST-SF3B1 (1–463) substrate. The phosphorylation was monitored using the indicated antibody.

To determine whether Thr595 in the T-loop is important for the kinase activity of CDK11^110^, we performed *in vitro* kinase assay (IVKA) with the recombinant N-terminal domain of SF3B1 and flag-tagged CDK11^110^ proteins immunopurified from HCT116 cells [32]. Wild-type CDK11^110^ efficiently phosphorylated the SF3B1 substrate, while a constitutively inactive mutation (T595A) lacked the kinase activity (Fig. 4A). Surprisingly, potentially phospho-mimicking mutations (T595E, T595D) also abolished the kinase activity (Fig. 4A). To determine whether Thr595 is important for the association of CDK11 with CCNL and SAP30BP, we immunoprecipitated CDK11^110^ proteins from cells and followed their association by western blotting. Wild-type CDK11 bound CCNL1, CCNL2, and SAP30BP efficiently, but any mutation of Thr595 resulted in a loss of interactions with those proteins (Fig. 4B and Supplementary Fig. S4A). We conclude that Thr595 in the T-loop of CDK11^110^ is essential for CDK11 kinase activity and CCNL1/L2 and SAP30BP association. It is worth noting that the results of the mutation experiments preclude an unequivocal conclusion as to whether the phosphorylation of the canonical Thr595 is needed for the regulation of the kinase activity and cyclin association, or whether this is just the result of the replacement of Thr with a different amino acid residue. However, considering the selective loss of CCNL1 and CCNL2 (relative to CDK11^110^) upon treatment with a CDK7 inhibitor (Fig. 3A), there is a strong, albeit circumstantial, case that the loss of phosphorylation is responsible for defects in CCNL/SAP30BP/CDK11 complex assembly.

The activating T-loop of CDK11 also bears a noncanonical Ser589 that is part of the Ser/Pro CDK consensus site (Fig. 2B). Ser in an analogous position (Ser164) is also present in the CDK7 T-loop, and its phosphorylation has been recently shown to promote the phosphorylation of canonical Thr170 in CDK7 [71] (Fig. 2B). To determine whether Ser589 is needed for association with CCNL and SAP30BP and for CDK11 kinase activity, we mutated this residue to constitutively inactive (S589A) or potentially phospho-mimicking (S589E and S589D) alternatives. The immunoprecipitation of S589A or S589E slightly, but reproducibly diminished the association with CCNL2 and SAP30BP (Fig. 4C). S589D did not cause any significant decrease in association, probably because in this context aspartic acid (D) functions as a phospho-mimicking mutant, whereas the larger glutamic acid (E) fails to do so. Correspondingly, a small decrease in SF3B1 phosphorylation in IVKAs was observed when using these mutants in comparison to wild-type CDK11 (Fig. 4D). These experiments suggest that Ser589 in the T-loop also contributes to the association of CDK11 with CCNL and SAP30BP, and therefore to the regulation of CDK11 kinase activity.

In summary, the data demonstrate that the T-loop of CDK11, and particularly Thr595, is vital for the formation of an active CDK11/CCNL/SAP30BP complex and CDK11 kinase activity.

### Onset of splicing defect after CDK7 inhibition correlates with P-CDK11^220^ dephosphorylation

**Figure 5.**
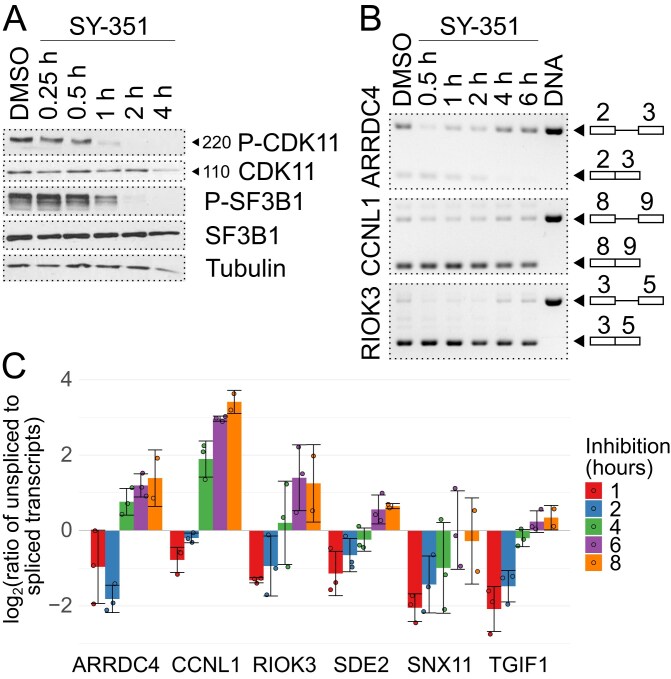
Onset of splicing deficiency after CDK7 inhibition correlates with P-CDK11^220^ dephosphorylation. (**A**) Immunoblot analyses of proteins after treatment of HCT116 cells with control DMSO or 50 nM SY-351 for the indicated times. Bands corresponding to CDK11^110^ and P-CDK11^220^ are marked on the right with arrows. (**B**) DNA gel-visualised RT-PCR analyses of splicing of *ARRDC4, CCNL1*, and *RIOK1* transcripts after treatment of HCT116 cells with 100 nM SY-351 for the indicated times. Schema of unspliced and spliced transcripts, including order of the tested exons in each transcript, are depicted on the right. DNA represents control RT-PCR product from genomic DNA. (**C**) Graph shows ratio of unspliced to spliced transcripts of six genes measured by RT-qPCR in HCT116 cells treated with 50 nM SY-351 for indicated times. mRNA levels were normalised to *PPIA* mRNA and expression in control DMSO condition was set as 1. Error bars = SD, *n* = 3.

Inhibition of CDK11 results in a quick dephosphorylation of SF3B1 (within 5 min), and an accumulation of unspliced introns is detected within 45 min [32]. One-hour CDK7 inhibition with SY-531 also produced a dephosphorylation of SF3B1, and a widespread splicing deficiency was reported after 5 h [11]. Considering the rapid effect of CDK11 inhibition on splicing, we wanted to determine whether the observed CDK7-dependent splicing abrogation might be a consequence of CDK11 inactivation. To compare the dynamics of CDK11^220^ and SF3B1 dephosphorylation with the onset of the splicing deficiency, we performed time-course experiments with SY-351 (Fig. 5). Western blot analyses revealed that the abundance of P-CDK11^220^ and P-SF3B1 started to decrease within 0.5–1 h after the start of the treatment, and the phosphorylation of both proteins completely disappeared within 2 h (Fig. 5A). This indicated that the phosphorylation of SF3B1 was retained until CDK11 was deactivated by dephosphorylation of its T-loop. RT-PCR on introns of selected genes showed an initial (within 0.5–1 h) decrease in nascent (unspliced) RNA accompanied by no change in the amounts of the spliced product (Fig. 5B). This can be attributed to the CDK7-mediated inhibition of transcription [10, 11, 18]. The accumulation of unspliced transcripts at the expense of the spliced ones only started to be detectable after 2 h of inhibition (Fig. 5B). The same trend was evident with RT-qPCR using primers covering exon–intron junctions in six genes (Fig. 5C). Comparison of RT-(q)PCR and immunoblot experiments revealed that the accumulation of unspliced transcripts coincided with the loss of P-CDK11^220^ (Fig. 5A–C). In contrast, the inhibition of CDK11 with OTS964 led to an immediate decrease in P-SF3B1 and accumulation of unspliced transcripts within 1 h of the treatment (Supplementary Fig. S5A and B), as reported previously [32]. Taken together, these data indicate that the reported splicing deficiency upon CDK7 inhibition is likely due to a decrease in P-CDK11^220^ and subsequent dephosphorylation of SF3B1 and spliceosome inactivation.

Phospho-proteomic screens coupled to the inhibition of other transcriptional CDKs (CDK9 and CDK12) identified SF3B1 as a potential substrate [72–74]. CDK9 regulates promoter-proximal pausing and its inhibition results in stalling of RNAPII at promoters of protein-coding genes leading to a global block of transcription [2, 75–77]. CDK12 regulates optimal RNAPII elongation and processivity and its inhibition results in slower elongation and premature termination, predominantly in long genes [72, 78–82]. Therefore, we aimed to determine whether inhibition of these CDKs affects SF3B1 phosphorylation and the formation of P-CDK11^220^. Cells were treated with 50 nM CDK9 inhibitor NVP-2 [83] or 100 nM CDK12/13 inhibitor THZ531 [84, 85] for 2 h and cellular fractions were analyzed as in Fig. 3A. CDK9 inhibition resulted in the loss of both P-SF3B1 and P-CDK11^220^ from chromatin (Supplementary Fig. S5C), a result similar to that observed upon CDK7 inhibition (Fig. 3A), and is consistent with transcriptional shutdown [2, 75, 76] and the consequent absence of assembled spliceosomes on chromatin [3]. CDK12 inhibition resulted in only a slight loss of P-SF3B1 and P-CDK11^220^ from the chromatin (Supplementary Fig. S5C), consistent with ongoing transcription and co-transcriptional splicing (i.e. formation of active spliceosomes on chromatin) [72, 80, 81]. To compare splicing defects following inhibition of CDK9 or CDK12 to inhibition of CDK7, we performed a time-course RT-PCR experiment as in Fig. 5B. After CDK9 inhibition, RT-PCR on introns of selected genes showed an initial (within 0.5–1 h) decrease in nascent (unspliced) RNA, accompanied by no change in the levels of the spliced products (Supplementary Fig. S5D). This result is comparable to that of CDK7 inhibition (Fig. 5B) and can be attributed to CDK9-mediated inhibition of transcription. Some accumulation of unspliced transcripts was observed after 2 and 4 h of CDK9 inhibition, respectively (Supplementary Fig. S5D); however, in contrast to CDK7 inhibition (Fig. 5B), a decrease in the levels of spliced transcripts at these later time points was not observed (compare Supplementary Fig. S5D to Fig. 5B). We interpret these changes upon longer-term CDK9 inhibition as a compensatory onset of nascent transcription, accompanied by ongoing active splicing. In contrast, CDK12 inhibition resulted in only marginal or no accumulation of unspliced transcripts after 4 h, accompanied by no decrease in spliced RNA (Supplementary Fig. S5D). These data are consistent with a minor loss of P-SF3B1 and P-CDK11^220^ from chromatin (Supplementary Fig. S5C) and the absence of a global splicing defects after CDK12 inhibition [72, 78, 80–82, 86–88].

### Core splicing factors identified as CDK7 substrates are also targets of CDK11

**Figure 6.**
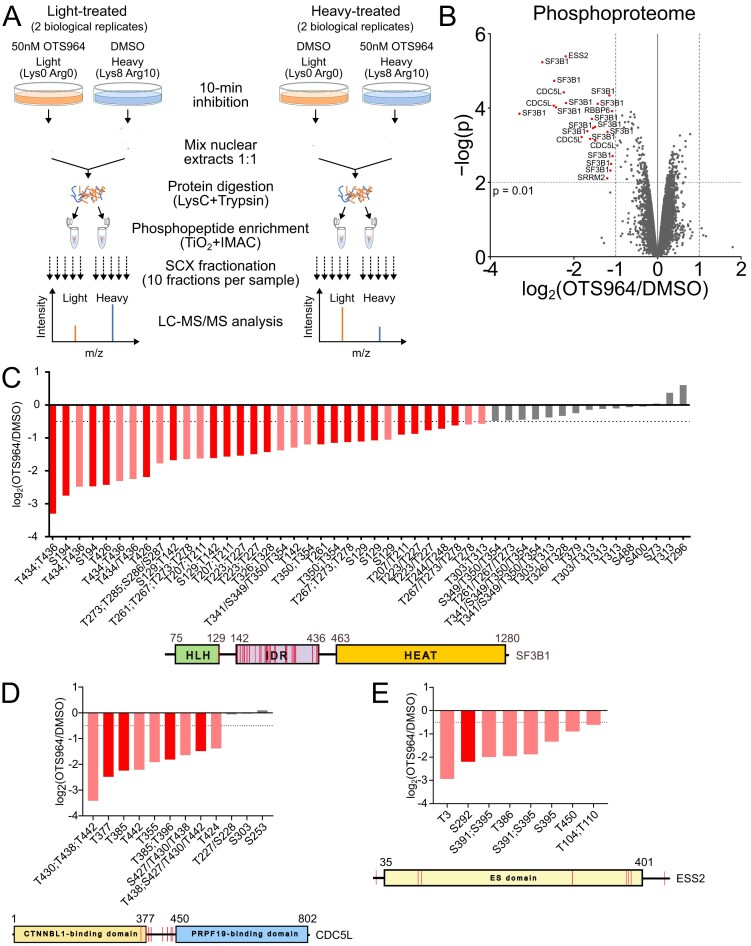
Identification of CDK11 substrates by SILAC phosphoproteomics. (**A**) Overview of design and workflow of SILAC labeling and phosphoproteomics experiment. HCT116 cells were metabolically labeled with “light” (Lys0 and Arg0) and “heavy” (Lys8 Arg10) medium and were treated with either DMSO or 50 nM OTS964 for 10 min. “Light” and “heavy” labeling was flipped for both treatments as indicated. The experiment was performed in biological duplicates. The cells were lysed and nuclear extracts were mixed in a 1:1 ratio. The proteins were digested by LysC and trypsin, and the digested phosphopeptides were enriched by immobilized metal affinity chromatography (IMAC) using titanium dioxide beads. The enriched phosphopeptides were separated into 10 fractions per sample by SCX chromatography. The phospho-peptides were identified by mass spectrometry, and light/heavy SILAC ratios were quantified to assess phosphorylation changes. (**B**) Volcano plot showing changes in protein phosphorylation after treatment of HCT116 cells with 50 nM OTS964 for 10 min. Statistically significantly decreased phosphopeptides of the indicated proteins (*P *< 0.01; log_2_ fold change < -1) are marked in red. (**C**–**E**) Graphs represent phosphopeptides identified in at least two replicates for splicing factors SF3B1 (C), CDC5L (D), and ESS2 (E) ordered according to log_2_ fold-change in dephosphorylation after treatment of HCT116 cells with 50 nM OTS964 for 10 min. Red bars = statistically significant decreased phosphorylation (*P *< 0.01; log_2_ fold-change < −0.5), pink bars = decreased phosphorylation (log_2_ fold-change < −0.5), gray bars = no change in phosphorylation (log_2_ fold-change > −0.5). The positions of phosphosites with decreased phosphorylation in SF3B1, CDC5L, and ESS2 are indicated by red or pink lines in the protein depictions below the graphs. The numbers above the protein depictions show amino acid positions bordering known protein domains (shown as colored squares). In SF3B1, HLH corresponds to helix-loop-helix, IDR to intrinsically disordered region and HEAT to HEAT-repeats domains.

CDK7 cellular substrates were recently identified after a short (1 h) inhibition with SY-531, suggesting that these proteins are primary targets of CDK7. Close inspection of the candidate substrates revealed Thr595 in CDK11 as one of the high-confidence targets, consistent with CDK11 being a downstream target of CDK7 [11]. Considering the recent discovery of extremely rapid dynamics of SF3B1 dephosphorylation and onset of splicing deficiency upon CDK11 inhibition [32] (and data shown here), we reasoned that some of the CDK7 splicing-related substrates found in recent screen [11] may in fact be an indirect consequence of CDK7-mediated CDK11 inactivation. It is worth noting that, with the exception of one Ser and ten Thr located in the SF3B1 N-terminus [3, 32], other CDK11 substrates remain unknown. We thus performed quantitative SILAC phospho-proteomics in cells treated with 50 nM OTS964 for 10 min (Fig. 6). The experimental design included two biological replicates in which HCT116 cells grown in “light” (Lys0 Arg0) medium were inhibited and cells grown in “heavy” medium (Lys8 Arg10) were treated with DMSO, and the labeling was flipped between the inhibited and control conditions (Fig. 6A). This “label-flip” ensures that any systematic errors that may arise due to labeling are eliminated. In total over 30 000 phosphosites were detected, with 12 701 phosphosites present in all four replicates (Supplementary Table S2). After CDK11 inhibition, 26 phospho-sites in five proteins (SF3B1, CDC5L, ESS2, SRRM2, and RBBP6) decreased significantly (log2FC ≤ -1, *P* value < 0.01) (Fig. 6B, and Supplementary Fig. S6A and Supplementary Table S2). The change in phosphosite abundance was not due to the changes in total protein levels, since no protein changed significantly within the timeframe of the experiment (Supplementary Fig. S6B). Importantly, no phosphosite was increased significantly, suggesting the absence of compensatory mechanisms after 10 min of the OTS964 treatment (Fig. 6B), and indicating that we only detected direct CDK11 substrates. To identify CDK11’s consensus phosphorylation motifs, we analyzed 26 phospho-sites that were significantly reduced upon the treatment. We found the presence of Thr and some Ser adjacent to Pro in position + 1 (Supplementary Fig. S6C), which corresponds to the minimal consensus motif for CDKs [89]. In SF3B1, sixteen Thr/Pro and two Ser/Pro phosphosites located in its N-terminus decreased statistically significantly (Fig. 6C). Additionally, the phosphorylation of another four Thr/Pro and one Ser/Pro sites were reduced (Fig. 6C), strongly suggesting that CDK11 phosphorylates most, if not all 28 Thr/Pro and some Ser-(Pro) sites in the the N-terminus of SF3B1. Splicing factors CDC5L and ESS2 (DGCR14) are newly identified CDK11 substrates (Fig. 6B and Supplementary Fig. S6A). Specifically, in CDC5L five phosphosites were statistically significantly decreased and an additional three ones were also reduced. All the sites are located in the central IDR rich in Thr/Pro motifs (Fig. 6D). In ESS2, the phosphorylation of Ser292 was significantly reduced, and an additional seven sites located across the protein were also dephosphorylated (Fig. 6E). Plotting the change in phosphorylation of all unique phosphopeptides identified in the SILAC experiment for SF3B1, CDC5L, ESS2, SRRM2, and RBBP6 showed that the total phosphorylation states of SF3B1, CDC5L, and ESS2 are largely dependent on CDK11 (Supplementary Fig. S6D). This suggests that CDK11 plays a more complex role in the regulation of pre-mRNA splicing.

Notably, the majority of the SF3B1, CDC5L and ESS2 phospho-sites detected here (Supplementary Fig. S6A and Supplementary Table S2) were also found in a phosphoproteomic screen of CDK7 substrates [11]. Despite the shorter CDK11 inhibition time (10 min here versus 60 min in the previous report [11]), it resulted in a stronger decrease in phosphorylation (compare Supplementary Table S2 with Table 1 in [11]). The direct effect of CDK11 in splicing was recently documented by the blockage of splicing and the spliceosome assembly in *in vitro* splicing assays [32]. In contrast, we did not observe any inhibition of the spliceosome assembly in *in vitro* splicing assays with high (10 µM) concentrations of SY-351 (Supplementary Fig. S6E), further supporting the conclusion that CDK7 affects pre-mRNA splicing in cells indirectly. In summary, we conclude that the splicing factors SF3B1, CDC5L, and ESS2 are direct substrates of CDK11, and CDK7 likely acts indirectly on them, i.e. via CDK11 to regulate their phosphorylation and pre-mRNA splicing.

## Discussion

We propose that a small fraction of CDK11 is phosphorylated at Thr595 within its T-loop, and is a component of the chromatin-bound active spliceosome in cells. This phosphorylated form of CDK11, referred here to as P-CDK11^220^, is likely a denaturing condition-resistant CDK11 dimer. We propose that P-CDK11^220^ represents the active form of the kinase in the spliceosome, that is responsible for phosphorylating SF3B1, thereby activating the spliceosome, and facilitating splicing. The formation of both P-CDK11^220^ and the activated spliceosome in cells are dependent on CDK7 kinase. CDK7 is in close proximity to CDK11, as revealed by the CDK11 Bio-ID assay reported here. Our data show that phosphorylatable residues in CDK11’s T-loop, particularly Thr595 (and to a lesser extent Ser589), are vital for the formation of the active CDK11/CCNL/SAP30BP complex. Additionally, using the selective CDK11 inhibitor OTS964 and quantitative phosphoproteomics, we identified novel CDK11 substrates, including a component of the spliceosome’s nineteen (NTC) complex, CDC5L protein [90], and the little-studied spliceosome protein ESS2 [91]. These splicing factors, and SF3B1, were also identified as CDK7 substrates in a previous study [11]. We propose that CDK7 indirectly regulates pre-mRNA splicing via CDK11, and that CDK11 plays a more complex role in the spliceosome regulation by phosphorylating other spliceosome components.

The inhibition of CDK11 with OTS964 causes a rapid (within 5 min) dephosphorylation of SF3B1 and intron retention within 1 h [32]. In contrast, the inhibition of CDK7 with SY-351 leads to dephosphorylation of the CDK11^220^ T-loop and SF3B1 within 1 to 2 h, and unspliced introns start to accumulate in 2–4 h. These findings suggest that the effects previously attributed directly to CDK7 [11] are likely due to CDK11 Thr595 dephosphorylation and subsequent rapid spliceosome inactivation. While creating constitutively active or inactive mutants of Thr595 would be useful to study the role of Thr595 phosphorylation, mutations at this site (e.g. Asp, Glu for activation to mimick phosphorylation or Ala for inactivation to block phosphorylation) disrupt kinase activity by preventing the formation of the active CDK11 complex. Since we failed to isolate or pre-form P-CDK11^220^  *in vitro*, we cannot conclusively separate the effects of Thr595 mutation vs. phosphorylation on the activity of CDK11.

Notably, CDK7 inhibition in a CDK7^AS/AS^ cell line with the competitive ATP analog 3MB-PP1 produced a lesser reduction of the level of P-CDK11^220^ compared to inhibition with the covalent inhibitor SY-351. This suggests a more complete inhibition of CDK7 activity with the covalent inhibitor, though different levels of CDK7 inhibition in different complexes (e.g. TFIIH and free CAK) cannot be ruled out.

Accumulating evidence shows that different CDKs have varying requirements for T-loop phosphorylation to associate with their cyclin subunits. For example, T-loop phosphorylation is not required for the corresponding cyclin binding in CDK2 [9], CDK4/6 [12], CDK9 [10], and CDK10 [92]. However, T-loop phosphorylation is essential for cyclin B binding to CDK1 [9], and for cyclin H interaction with CDK7 in the absence of the scaffolding protein MAT1 [93]. In case of CDK11, we could not distinguish between the phosphorylated and nonphosphorylated T-loops, nevertheless we found Thr595 to be necessary for the formation of the active CDK11/CCNL/SAP30BP complex. CDK11 is phosphorylated at Thr595 within what appears to be a dimer (P-CDK11^220^) in the context of the activated spliceosome, suggesting that a distinct activation mechanism via T-loop phosphorylation exists. The precise molecular and structural details of this mechanism remain unclear and warrant further investigation.

We attempted to test whether CDK7 can activate CDK11 kinase activity *in vitro* using the CDK7 ternary complex (CDK7/CCNH/MAT1) from insect cells, the analog-sensitive (AS) CDK11^110^ complex isolated from human cells, and N-terminal SF3B1 purified from bacteria. However, we did not observe the formation of P-CDK11^220^ under these conditions (data not shown). This result may be explained by the fact that P-CDK11^220^ likely only forms within the context of the activated spliceosome complex on chromatin in cells. Phospho-proteomic analysis after one hour of treatment with SY-351 revealed a diminished phosphorylation of CDK11 at Thr595 in cells [11]. The previous study by Larochelle *et al.* has shown that CDK7 can phosphorylate Thr but not Ser in the activation loop of the CDK11^58^ isoform *in vitro* [67]. Since we and others [33, 41, 42] did not detect CDK11^58^ expression in HCT116 or other cell lines, the existence and function of this isoform remain unclear.

Time-course experiments show that CDK7 inhibition leads to an immediate decrease in global transcription, as a lack of CTD phosphorylation causes initiation and Mediator factors to remain bound to RNAPII at gene promoters and during early elongation. Compensatory phosphorylation by other transcriptional kinases (CDK9/12/13) further downstream in gene bodies leads to the release of initiation/Mediator factors and the recruitment of elongation factors, accelerating transcription [11, 18, 22]. This sequence of events is reflected in our RT-(q)PCR experiments, which show an initial reduction of nascent transcripts within 1 h of CDK7 inhibition, followed by a rescue of transcription at later time points. This results in the accumulation of unspliced pre-mRNAs starting after 2 h, which reflects ongoing CDK7 inhibitor-mediated inactivation of the spliceosome. Thus, while the CDK7 inhibition of transcription can be at least partially rescued by the above mechanism [11, 18, 22], the CDK7-mediated spliceosome inactivation seems to not be compensated by other kinases, at least within the time-frame of our experiment.

Notably, inhibition of other transcriptional kinases, CDK9 and CDK12, also reduced SF3B1 phosphorylation and the formation of P-CDK11^220^ (see Supplementary Fig. S5C). This result is consistent with outcomes of several phospho-proteomic screens after CDK9 or CDK12 inhibition [72, 74] and likely reflects roles of these kinases in the process of transcription, which subsequently determines spliceosome assembly and thus SF3B1 phosphorylation [3]. Indeed, the effects on levels of unspliced (nascent) and spliced RNAs for the tested introns in time-course RT-PCR experiments varied depending on the kinase (CDK7, CDK9, and CDK12/13) (see Fig. 5B and Supplementary Fig. S5D). At early time points (<2 h), a reduction in unspliced RNA reflected each kinase’s ability to shut down transcription and thereby limit nascent RNA production. This effect was observed only with inhibition of CDK7 and CDK9, as expected [2, 10, 11, 18, 75]. At later time points (>2 h), the levels of unspliced and spliced RNA likely reflect the activation of distinct compensatory mechanisms triggered by inhibition of each kinase. Importantly, only CDK7 inhibition led to an increase in levels of unspliced RNAs at the expense of spliced ones (compare Fig. 5B with Supplementary Fig. S5D). In agreement, direct inhibition of pre-mRNA splicing by OTS964 or pladienolide B results in an immediate accumulation of unspliced introns at the *expense* of spliced ones across all tested time points (0.5–8 h) (see Supplementary Fig. S5B and [32]). These observations support our model in which CDK7 regulates pre-mRNA splicing through activation of CDK11.

SF3B1 and CDC5L are conserved splicing factors that are also found in yeast [94], but their intrinsically disordered Thr/Pro-rich regions, phosphorylated by CDK11, are only present in higher eukaryotes. ESS2, which is also phosphorylated by CDK11, is only present in higher eukaryotes and is absent in *Saccharomyces cerevisiae*. This suggests that CDK11 and its substrates have co-evolved within the spliceosome, reflecting the more need for spliceosome regulation due to the increased complexity of pre-mRNA splicing in higher eukaryotes compared to yeast [3, 29]. The regulatory roles of the CDK11-mediated phosphorylation of CDC5L and ESS2 in splicing remain to be determined.

Finding that additional CDK11 substrates are predominantly core spliceosome components provides further support for the hypothesis that CDK11 is essential for cell viability and proliferation in higher eukaryotes due to its key role in the process of splicing, that is tightly linked with cell cycle progression via a feedback loop [3, 95–97]. Likewise, the inhibition of spliceosome activation and splicing following CDK7 inhibition (Figs 3A and B, and 5B and C) raises the question of to what extent splicing deficiency contributes to CDK7-dependent cell cycle progression deficiencies. While CDK7 is known to activate cell cycle CDKs through its CAK activity in metazoans [9, 12, 14], and regulate subset of core transcription factors that control cell cycle and proliferative gene expression programs [98], the underlying mechanism may be more complex, possibly involving the deregulation of the CDK7–CDK11 axis and its impact on spliceosome function. Further investigation of the contribution of individual CDK7 pathways to cell cycle progression is needed. This will be of interest, since CDK7 and CDK11 have been identified as potential drug targets for anticancer therapy [2, 27, 50, 51].

It is also worth noting that it is only in metazoans where CDK11 regulates pre-mRNA splicing and is essential for cellular proliferation. CDK11 is absent in *S. cerevisiae*, and in *Schizosaccharomyces pombe* it regulates the transcription of a subset of genes and is nonessential [3, 99]. Likewise, in metazoans CDK7 in the free CAK complex (e.g. independently of its TFIIH function) is essential for cell cycle regulation via the phosphorylation and activation of cell cycle CDKs [13, 14]*. S. pombe* CDK7 ortholog *mcs6* in CAK complex is needed for the expression of growth-related genes via activation of cdc2 [14, 100]. Mutations in CDK7 *S. cerevisiae* ortholog *kin28*, neither cause cell cycle arrest nor CDK activation deficiency as the kinase is devoid of CAK activity [14].

**Figure 7.**
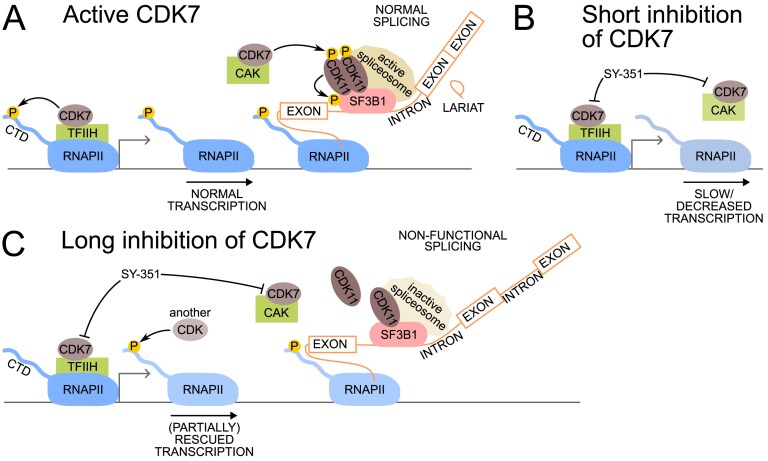
Working Model. (**A**) CDK7 activity in normal cell. CDK7, as part of the TFIIH complex, phosphorylates the CTD of RNAPII to regulate promoter escape and normal transcription. CDK7, probably as part of the CAK complex, is also required for the onset of P-CDK11^220^, which is needed for the phosphorylation of SF3B1 (P-SF3B1) and activation of the spliceosome and normal splicing. (**B**) Consequences of short (<2 h) CDK7 inhibition with SY-351. Short inhibition blocks promoter escape, which results in a strong decrease in transcription, with some RNAPII slowly transcribing into the gene body. (**C**) Consequences of long (>2 h) CDK7 inhibition with SY-351. After a longer inhibition, the transcription is rescued by other transcriptional CDKs that phosphorylate the CTD of RNAPII [18]. This results in faster elongation and synthesis of nascent RNA. The RNA is not spliced, because ongoing CDK7 inhibition blocks the formation of P-CDK11^220^ and subsequent SF3B1 phosphorylation, which results in an inactive spliceosome and nonfunctional splicing.

Based on our studies and recent work on CDK7’s role in transcription [10, 18], we propose the following working model (Fig. 7): CDK7 is needed for the formation of P-CDK11^220^, which results in the phosphorylation of SF3B1 and activation of the spliceosome. The inhibition of CDK7 initially (<2 h) blocks transcription, which is later (>2 h) rescued by a compensatory mechanism [11, 18, 22]. The newly synthesized transcripts, however, are not spliced, due to the continued CDK7-mediated inactivation of the spliceosome. This leads to the accumulation of unspliced transcripts in cells after longer (>2 h) CDK7 inhibition (Fig. 5B and C).

### Limitations of the study

Our observation that CDK11, phosphorylated at Thr595, forms a denaturation-resistant dimer within the activated spliceosome complex requires further investigation. We hypothesize that this subset of CDK11 has a unique propensity for self-aggregation to dimers within the spliceosome, which may serve a regulatory function to enable its activation in this context. The experiments using total and phospho-specific CDK11 antibodies reported herein strongly indicate that CDK11^220^ exists only in the form that is phosphorylated at Thr595, but we cannot conclude whether the phosphorylation is a cause or consequence of CDK11^220^ formation. We believe that mechanism(s) of CDK11 activation in other cellular complexes may be different. We confirmed the dimerization of CDK11^110^ in cells (see Supplementary Fig. S2E), but we did not identify the phosphorylation of Thr595 in CDK11^110^ form by several phospho-specific antibodies. This suggests that the dimerization alone is not a sufficient pre-condition for the occurrence of P-Thr595 and also CDK11^220^ formation. CDK7-mediated phosphorylation of CDK11 was initially observed in CDK11^110^ and mapped to the canonical activating Thr in the T-loop of recombinant CDK11^58^ [67]. However, we found that CDK11^58^ is not expressed in HCT116 cells (Supplementary Fig. S2F and G), and Thr595 is not phosphorylated in CDK11^110^ using several P-Thr595-specific antibodies (Supplementary Fig. S2B). Further experiments are needed to resolve this discrepancy.

Although CDK7 was found to be in close proximity to CDK11 in the proximity assay, and the two kinases interact in the presence of the CDK7 inhibitor, we cannot exclude the possibility that the observed decrease in SF3B1 phosphorylation is, at least partly, a consequence of a general transcriptional block caused by CDK7 inhibition. However, unequivocally distinguishing between these scenarios is a challenging task, as transcription is an upstream process of splicing. Therefore, its inhibition inherently disrupts spliceosome assembly and, consequently, SF3B1 phosphorylation [3, 101] (see also Discussion).

Also, we cannot exclude the possibility that additional CDK7 substrates [11, 18, 67] contribute to the CDK7-mediated effects on splicing. Since most of the experiments were conducted only in a single cell line, we cannot exclude the possibility that some differences between our results and published data could be affected by the choice of particular cell line.

## Supplementary Material

gkaf1343_Supplemental_Files

## Data Availability

All next-generation-sequencing source and processed data are available at NCBI GEO (accession number: GSE290846). Mass spectrometry proteomics data were deposited to the ProteomeXchange Consortium via the PRIDE [102] partner repository with the following dataset identifiers: PXD068896 – BioID dataset, PXD062535 – SILAC-MS dataset.
