# Supplementary material for: CDK7–CDK11 axis in spliceosome regulation and pre-mRNA splicing
Source: Nucleic Acids Res. 2025 Dec 22;53(22):gkaf1343. doi: 10.1093/nar/gkaf1343 (PMC12721332; doi:10.1093/nar/gkaf1343)
Supplement: gkaf1343_Supplemental_Files [file gkaf1343_supplemental_files.zip › Supplementary.pdf]

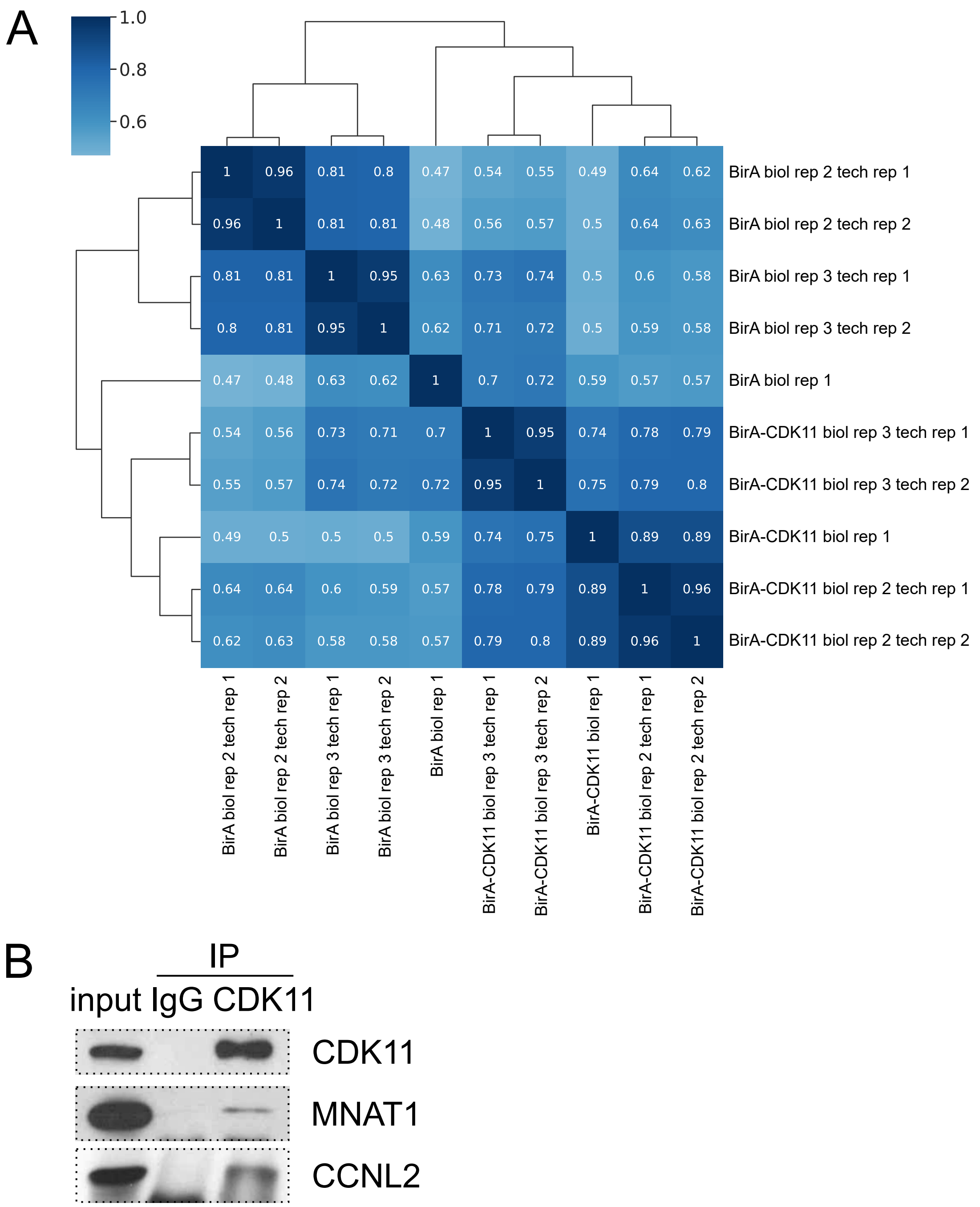

Supplementary Figure 1. Correlation of BioID replicates. (**A**) Correlation analyses between indicated biological replicates of control BirA and BirA-CDK11 samples. Numbers correspond to R^2 (Pearson correlation coefficient) between the indicated replicates, and is also indicated by the colour code. (**B**) Immunoblot analysis of immunoprecipitations of endogenous CDK11 after 4-h treatment with 50 nM SY-351 in HCT116 cells. Detected proteins are indicated on the right. IgG = antibody control.

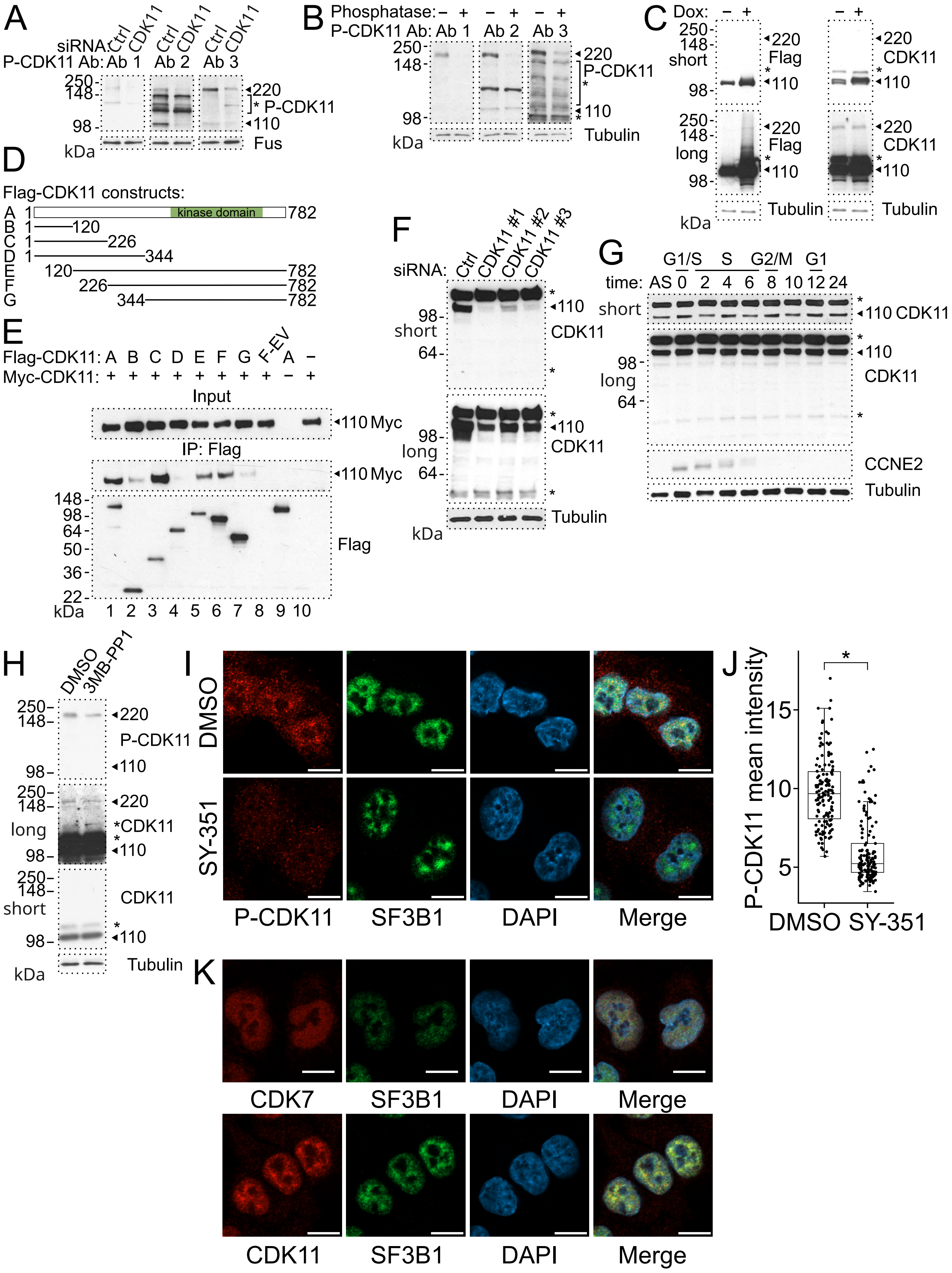
A
siRNA: Ctrl CDK11 Ctrl CDK11 Ctrl CDK11
P-CDK11 Ab: Ab 1 Ab 2 Ab 3
250
148
98
kDa
220
* P-CDK11
110
Fus
B
Phosphatase: – + – + – +
P-CDK11 Ab: Ab 1 Ab 2 Ab 3
250
148
98
kDa
220
P-CDK11
*
110
Tubulin
C
Dox: – + – +
250
148
short
98
220
Flag
110
CDK11
long
Tubulin
kDa
D
Flag-CDK11 constructs:
A 1 kinase domain 782
B 1 120
C 1 226
D 1 344
E 120 782
F 226 782
G 344 782
E
Flag-CDK11: A B C D E F G F-EV A –
Myc-CDK11: + + + + + + + + – +
Input
110 Myc
IP: Flag
148
98
64
50
36
22
kDa
Flag
1 2 3 4 5 6 7 8 9 10
F
siRNA: Ctrl CDK11 #1 CDK11 #2 CDK11 #3
98
short
64
long
110
CDK11
Tubulin
kDa
G
G1/S S G2/M G1
time: AS 0 2 4 6 8 10 12 24
short
110 CDK11
98
long
64
110
CDK11
CCNE2
Tubulin
H
DMSO 3MB-PP1
250
148
98
220
P-CDK11
110
long
CDK11
short
Tubulin
kDa
I
DMSO
SY-351
P-CDK11 SF3B1 DAPI Merge
J
P-CDK11 mean intensity
15
10
5
DMSO SY-351
K
CDK7 SF3B1 DAPI Merge
CDK11 SF3B1 DAPI Merge

Supplementary Figure 2. P-CDK11220 is likely a denaturing condition-resistant dimer. (**A,B**) Immunoblots with indicated phospho-specific CDK11 antibodies (Ab1, Ab2, Ab3) using HCT116 cell lysates treated with either control or CDK11 siRNAs for 42 h (A) or with alkaline phosphatase for 1 h (B). Bands corresponding to $CDK11^{110}$ and $P\text{-}CDK11^{220}$ are marked on the right with arrows. Asterisk denotes a non-specific bands. (**C**) Immunoblots with indicated antibodies using HCT116 cell lysate with flag-tagged CDK11 expression either induced or not with doxycycline (Dox). Bands corresponding to $CDK11^{110}$ and $CDK11^{220}$ are marked on the right with arrows. "Long" and "short" corresponds to long and short exposure of the film. Asterisk denotes a non-specific band. (**D**) Schema of flag-tagged CDK11 wild-type and deletion mutant plasmids used in experiments presented in Supp. Fig. 2E. (**E**) Immunoblot analyses of immunoprecipitations with flag antibody in HCT116 cell lysates co-expressing indicated combinations of flag-tagged CDK11 proteins and myc-tagged wild-type CDK11. F-EV represents flag-tagged empty vector. The band corresponding to $CDK11^{110}$ is indicated by the arrow. (**F**) Immunoblot analyses of HCT116 cell lysates treated with control or three different CDK11 siRNAs for 42 h. The band corresponding to $CDK11^{110}$ is indicated by the arrow, asterisk denotes a non-specific band. "Long" and "short" corresponds to long and short film exposure, respectively. (**G**) Immunoblot of indicated proteins in HCT116 cells synchronized in G1/S cell cycle phase by double-thymidine treatment (0 h). Time points after the release and cell cycle phases are indicated. AS corresponds to asynchronous cells. The band corresponding to $CDK11^{110}$ is indicated by the arrow, asterisk denotes a non-specific band.
h) Immunoblot of cell lysates from HCT116 $CDK7^{AS/AS}$ cells treated with either control DMSO or 40 µM 3MB-PP1 for 4 h. See Fig. 2D for the figure legend. (**I, J, K**) See Figs 2E, F for the figure legend. P-CDK11 was monitored by Ab1. CDK7 and CDK11 were monitored without inhibition.

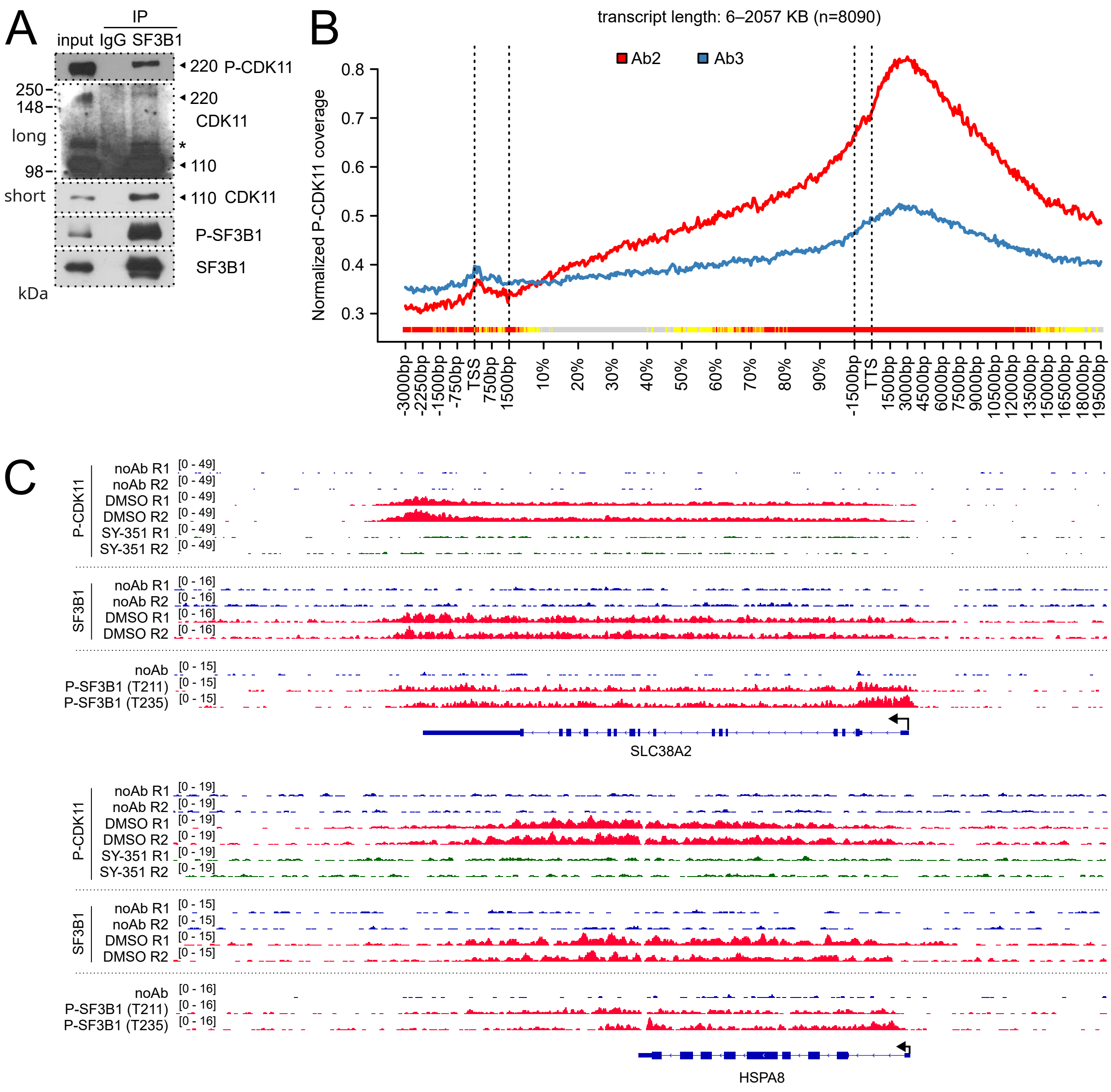

Supplementary Figure 3. P-CDK11^{220} is associated with chromatin-bound spliceosomes. (**A**) Immunoblot analyses of immunoprecipitations of endogenous SF3B1 from chromatin fraction in HCT116 cells. Detected proteins are indicated on the right. Bands corresponding to CDK11^{110} and P-CDK11^{220} are marked on the right with arrows. “Long” and “short” corresponds to long and short exposure of the film. Asterisk denotes a non-specific band. IgG, antibody control. (**B**) Comparison of metagene P-CDK11 ChIP-Seq profiles performed with Ab2 and Ab3. See Fig. 3C for the figure legend. (**C**) IGV genome browser view of P-CDK11^{220} ChIP-Seq on *SLC38A2* and *HSPA8* genes in HCT116 cells treated with either control DMSO or 50 nM SY-351 for 2 h. SF3B1 and P-SF3B1 ChIP-seq on *SLC38A2* and *HSPA8* genes in cells treated with control DMSO are shown below. noAb = control without antibody, R1, R2 = replicate 1, 2. T211 and T235 Ab=antibodies against P-Thr211 and P-Thr235 in SF3B1, respectively. Y-axis scale is denoted in square brackets.

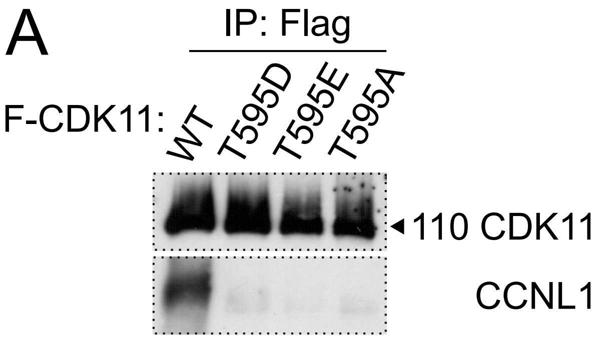

Supplementary Figure 4. Thr595 in CDK11 is essential for the formation of CDK11/CCNL complex. (**A**) Immunoblot analyses of immunoprecipitations of flag-tagged wild type (WT) or Thr595 mutants (T595A, T595D, T595E) from HCT116 cells. Detected proteins are indicated on the right.

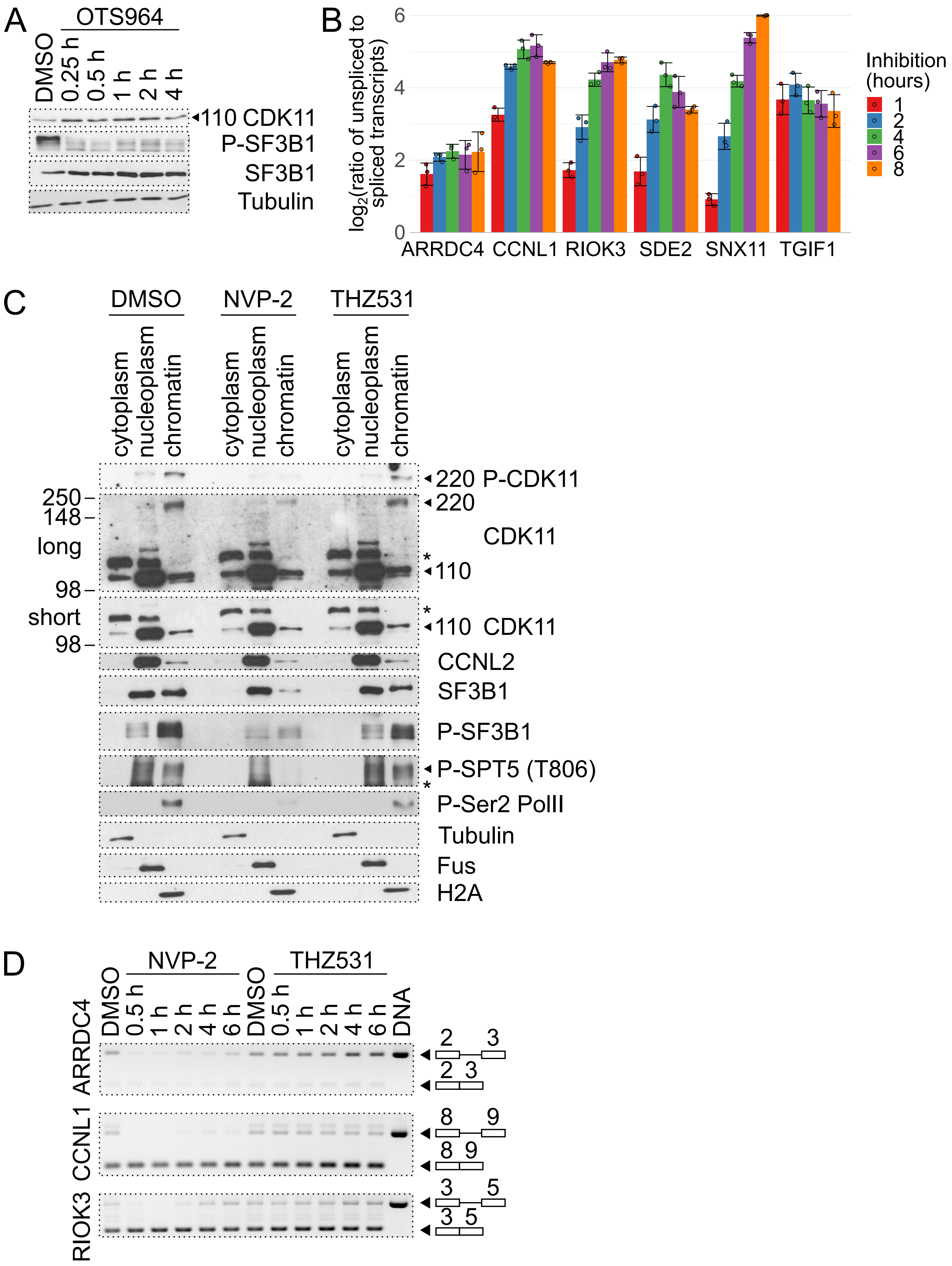
A
OTS964
DMSO
0.25 h
0.5 h
1 h
2 h
4 h
110 CDK11
P-SF3B1
SF3B1
Tubulin
B
log2(ratio of unspliced to spliced transcripts)
0
2
4
6
ARRDC4
CCNL1
RIOK3
SDE2
SNX11
TGIF1
Inhibition (hours)
1
2
4
6
8
C
DMSO
NVP-2
THZ531
cytoplasm
nucleoplasm
chromatin
220 P-CDK11
250
148
long
98
220
CDK11
*
110
short
98
*
110 CDK11
CCNL2
SF3B1
P-SF3B1
P-SPT5 (T806)
*
P-Ser2 PolII
Tubulin
Fus
H2A
D
NVP-2
THZ531
DMSO
0.5 h
1 h
2 h
4 h
6 h
DNA
ARRDC4
2
3
CCNL1
8
9
RIOK3
3
5

Supplementary Figure 5. CDK11 inhibition rapidly blocks splicing. (**A**) Immunoblot analyses of proteins after treatment of HCT116 cells with 50 nM OTS964 for the indicated times. Band corresponding to $CDK11^{110}$ is marked on the right with an arrow. (**B**) Graph shows ratio of unspliced over spliced transcripts of six genes measured by RT-qPCR in HCT116 cells treated with 50 nM OTS964 for the indicated times. mRNA levels were normalised to PPIA mRNA and expression in control DMSO condition was set as 1. Error bars=SD, n=3. (**C**) Immunoblot analyses of indicated proteins in HCT116 cells treated with control DMSO or 50 nM NVP-2 or 100 nM THZ531 for 2 h and separated into cytoplasmic (cyto), nucleoplasm (nucl) and chromatin (chrom) fractions. Bands corresponding to $CDK11^{110}$ and P-$CDK11^{220}$ (monitored by Ab3) are marked on the right with arrows. “Long” and “short” corresponds to long and short exposure of the film. Asterisk denotes a non-specific band. (**D**) DNA gel-visualised RT-PCR analyses of splicing of *ARRDC4*, *CCNL1* and *RIOK1* transcripts after treatment of HCT116 cells with 50 nM NVP-2 (left) or 100 nM THZ531 (right) for the indicated times. Schema of unspliced and spliced transcripts, including order of the tested exons in each transcript, are depicted on the right. DNA represents control RT-PCR product from genomic DNA.

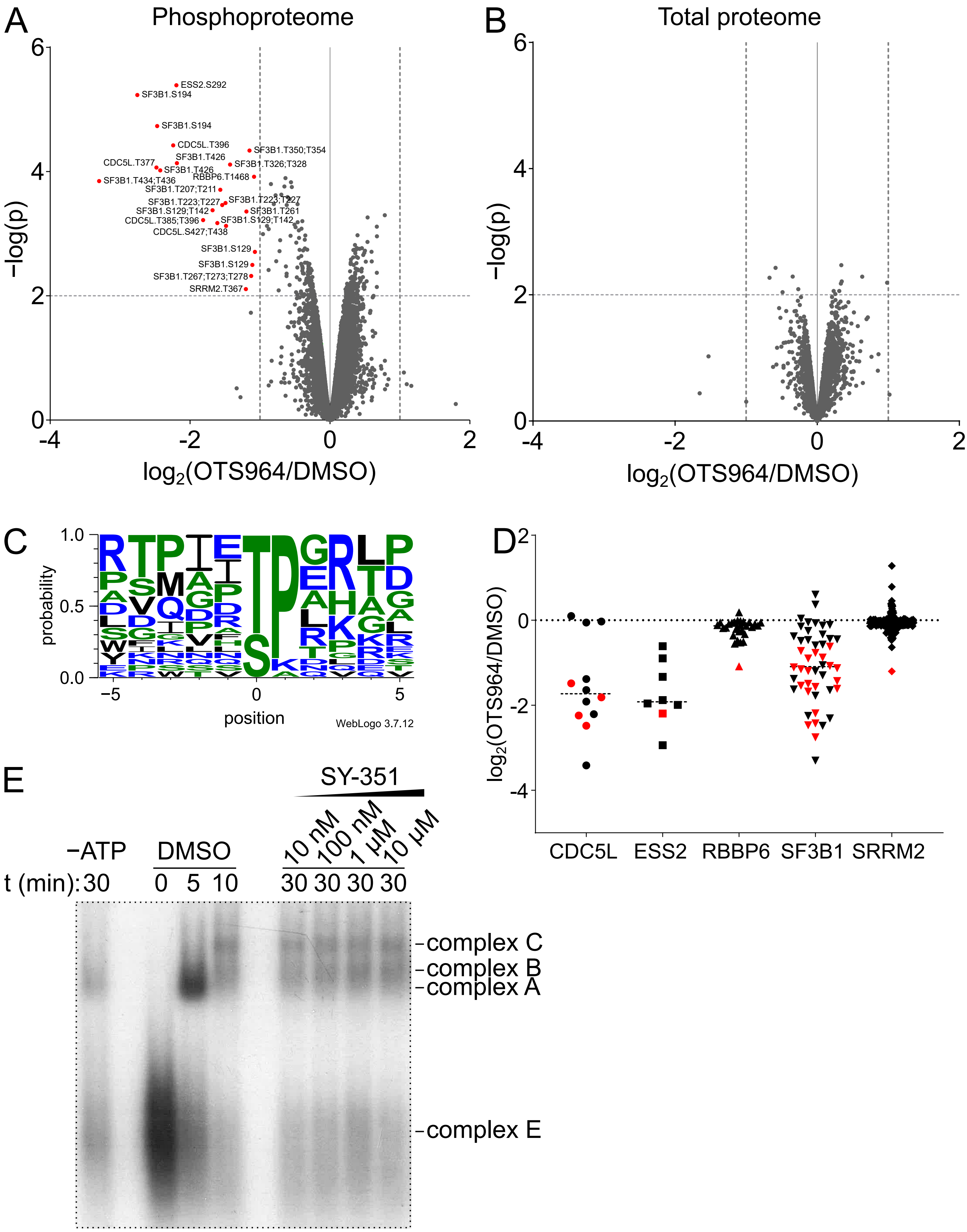
A
Phosphoproteome
B
Total proteome
−log(p)
log2(OTS964/DMSO)
ESS2.S292
SF3B1.S194
SF3B1.S194
CDC5L.T396
SF3B1.T350;T354
SF3B1.T426
CDC5L.T377
SF3B1.T326;T328
SF3B1.T426
RBBP6.T1468
SF3B1.T434;T436
SF3B1.T207;T211
SF3B1.T223;T227
SF3B1.T223;T227
SF3B1.S129;T142
SF3B1.T261
CDC5L.T385;T396
SF3B1.S129;T142
CDC5L.S427;T438
SF3B1.S129
SF3B1.S129
SF3B1.T267;T273;T278
SRRM2.T367
C
probability
position
WebLogo 3.7.12
D
log2(OTS964/DMSO)
CDC5L
ESS2
RBBP6
SF3B1
SRRM2
E
SY-351
−ATP
DMSO
10 nM
100 nM
1 µM
10 µM
t (min): 30
0 5 10
30 30 30 30
complex C
complex B
complex A
complex E

Supplementary Figure 6. CDK11 is predominantly Thr/Pro kinase. (**A**) Volcano plot showing changes in protein phosphorylation after treatment of HCT116 cells with 50 nM OTS964 for 10 min. See figure legend for Fig. 6B. Exact position of phosphorylation in the statistically significantly decreased phosphopeptides ($p < 0.01$; $\log_2$ fold change < −1) are shown. (**B**) Volcano plot of changes in total proteome after treatment of HCT116 cells with 50 nM OTS964 for 10 min. (**C**) Phosphorylation consensus sequence motifs for CDK11 derived by WebLogo 3.7.12 software from statistically significantly decreased phosphopeptides ($p < 0.01$; $\log_2$ fold-change < −1) after the treatment of HCT116 cells with 50 nM OTS964 for 10 min. The phosphorylation site detected by mass spectrometry is marked as position 0 in the motif. (**D**) Plot shows $\log_2$ fold-change in phosphorylation in phosphopeptides detected in at least two replicates in the CDC5L, ESS2, RBBP6, SF3B1 and SRRM2 proteins upon treatment of HCT116 cells with 50 nM OTS964 for 10 min. Each dot corresponds to one type of a phosphopeptide; the peptides with statistically significantly decreased phosphorylation ($p < 0.01$; $\log_2$ fold-change < −0.5) are marked in red. (**E**) Native gel analysis of spliceosome complex formation on radiolabelled *AdML* pre-mRNA in HeLa nuclear extracts treated with DMSO or indicated concentrations of SY-351 for indicated times. Identities of spliceosome complexes E, A, B and C are depicted on the side. −ATP corresponds to ATP-depleted nuclear extracts.

SUPPLEMENTARY TABLES AND LEGEND

Supplementary Table S1: Proteins identified in Bio-ID experiment

Supplementary Table S2: Proteins identified in SILAC phosphoproteomics experiment

Supplementary Table S3: List of antibodies used in this study

Supplementary Table S4: List of primers used in this study

Supplementary Table S5: List of plasmids used in this study

Supplementary Table S6: List of siRNAs used in this study

Supplementary Table S7: List of cell lines used in this study

SUPPLEMENTARY MATERIAL AND METHODS

Synthesis of SY-351

We prepared the compound SY-351 by synthesis that included modified final steps of the route published previously [1].

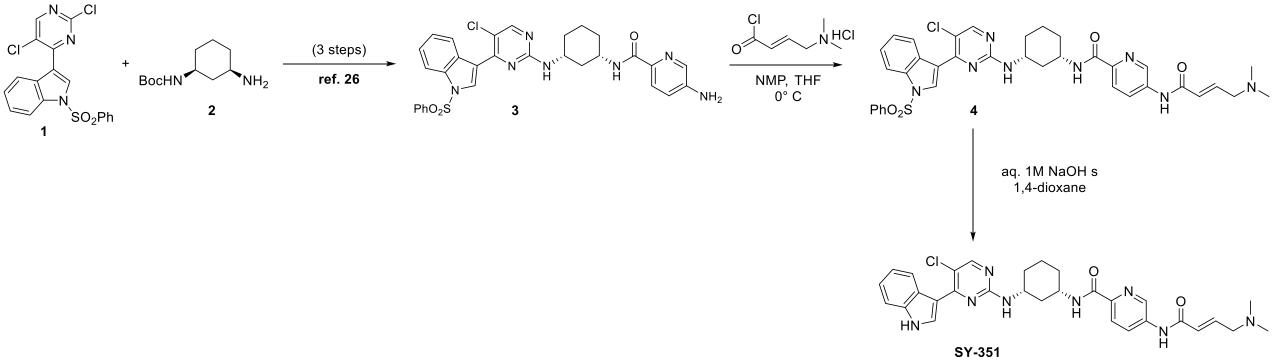

General Experimental Procedures. All reactions were performed in round-bottom flasks fitted with rubber septa under nitrogen atmosphere. Air and moisture-sensitive liquids were transferred by syringe. Analytical thin-layer chromatography (TLC) was performed using aluminum plates pre-coated with silica gel (silica gel 60 F_{254}, Merck). TLC plates were visualized by exposure to ultraviolet light (λ = 254 nm) and/or by submersion in aqueous ceric ammonium molybdate (CAM). All solutions were concentrated by rotary evaporation at 45 °C. Flash column chromatography was carried out on silica gel (60 Å, 230–400 mesh, Sigma-Aldrich). All reagents were obtained from commercial suppliers and were used without further purification. Anhydrous solvents were used from commercial suppliers (Sigma-Aldrich, Acros Organics) and stored over 4 Å molecular sieves.

Instrumentation. Nuclear magnetic resonance spectra were recorded using Bruker Avance 500 MHz instrument at 30 °C. Data are represented the following way: chemical shift, multiplicity (s = singlet, d = doublet, t = triplet, q = quartet, m = multiplet and/or multiple resonances), coupling constant (*J*) in Hz. Proton chemical shifts are expressed in parts per million (ppm, δ scale) and are referenced to residual H in the NMR solvents CHD_2SOCD_3, δ 2.50 ppm. Carbon chemical shifts are expressed in parts per million (ppm, δ scale) and are referenced to the carbon

resonances of the NMR solvents $(CD_3)_2SO$, δ 39.52 ppm. High-resolution mass spectra were obtained on Agilent 6224 Accurate-Mass TOF LC-MS with dual electrospray/chemical ionization mode or on MALDI-TOF Ultraflextreme (Bruker Daltonics) with positive ions detection.

5-amino-*N*-((1*S*,3*R*)-3-((5-chloro-4-(1-(phenylsulfonyl)-1*H*-indol-3-yl)pyrimidin-2-yl)amino)cyclohexyl)picolinamide (3):

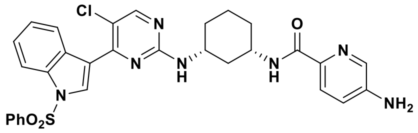

This compound was prepared according to the published procedure [1].

***N*-((1*S*,3*R*)-3-((5-chloro-4-(1-(phenylsulfonyl)-1*H*-indol-3-yl)pyrimidin-2-yl)amino)cyclohexyl)-5-((*E*)-4-(dimethylamino)but-2-enamido)picolinamide (4):**

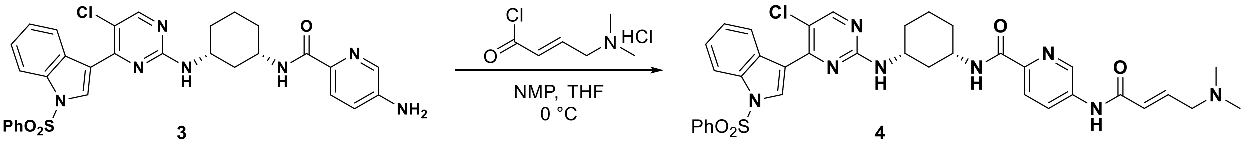

A suspension of (*E*)-4-(dimethylamino)but-2-enoyl chloride hydrochloride (178 mg, 0.965 mmol, 1.15 eq. supplier: Enamine Ltd, CAS: 1055943-40-2) in THF (8 mL) was added at 0 °C (ice/water bath) to a solution of 5-amino-*N*-((1*S*,3*R*)-3-((5-chloro-4-(1-(phenylsulfonyl)-1*H*-indol-3-yl)pyrimidin-2-yl)amino)cyclohex yl)picolinamide (505 mg, 0.839 mmol, 1 eq.) in *N*-methyl-2-pyrrolidone (6 mL). The reaction mixture was stirred at 0 °C for 90 min (TLC indicated full conversion), quenched with water (35 mL), and pH was adjusted to 11 by addition of 10% aqueous sodium hydroxide solution (ca. 40 mL). The reaction mixture was extracted with ethyl acetate (3 x 150 mL). The organic extracts were separated, dried over $MgSO_4$, filtered, and the solvent was evaporated *in vacuo*. The residue was purified by reversed phase flash column chromatography (water-methanol-7 M NH_3 in methanol, 0:100:2, Biotage, column: 12 g). The product **4** was obtained as a white solid (440 mg, 74%).

^{1}H NMR (500 MHz, dimethylsulfoxide-d_6) δ (ppm) 10.51 (s, 1H), 8.87 (d, J = 2.5 Hz, 1H), 8.61 (br s, 1H), 8.51 – 8.45 (m, 1H), 8.44 – 8.38 (m, 2H), 8.22 (dd, J = 8.6, 2.5 Hz, 1H), 8.12 – 8.06 (m, 2H), 8.02 – 7.96 (m, 2H), 7.75 – 7.70 (m, 1H), 7.65 – 7.60 (m, 2H), 7.57 – 7.54 (m, 1H), 7.48 – 7.40 (m, 2H), 6.81 (dt, J = 15.4, 5.8 Hz, 1H), 6.29 (dt, J = 15.4, 1.7 Hz, 1H), 3.97 – 3.80 (m, 2H), 3.08 (dd, J = 5.9, 1.7 Hz, 2H), 2.20 – 2.14 (m, 1H), 2.18 (s, 6H), 1.96 – 1.90 (m, 1H), 1.84 – 1.76 (m, 2H), 1.54 – 1.46 (m, 1H), 1.43 – 1.36 (m, 2H), 1.28 – 1.21 (m, 1H).

^{13}C NMR (126 MHz, dimethylsulfoxide-d_6) δ (ppm) 163.82, 162.53, 159.63, 144.71, 142.84, 138.96, 138.08, 136.44, 135.08, 133.68, 130.03, 129.41, 128.54, 126.97, 126.49, 125.55, 125.05, 122.44, 117.22, 112.90, 59.61, 49.26, 47.09, 45.10, 38.56, 31.42, 31.29, 22.98.

HRMS (APCI): calcd. for $C_{36}H_{38}ClN_8O_4S$ $[M+H]^+$ 713.2420, found 713.2416.

***N*-((1*S*,3*R*)-3-((5-chloro-4-(1*H*-indol-3-yl)pyrimidin-2-yl)amino)cyclohexyl)-5-((*E*)-4-(dimethylamino)but-2-enamido)picolinamide (SY-351):**

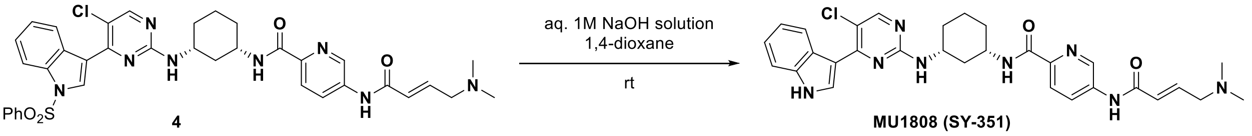

Aqueous 1M sodium hydroxide solution (5.04 mL, 5.05 mmol, 18 eq.) was added to a solution of *N*-((1*S*,3*R*)-3-((5-chloro-4-(1-(phenylsulfonyl)-1*H*-indol-3-yl)pyrimidin-2-yl)amino)cyclohexyl)-5-((*E*)-4-(dimethylamino)but-2-enamido)picolinamide (200 mg, 0.280 mmol, 1 eq.) in 1,4-dioxane (4 mL), and the resulting mixture was stirred at 23 °C for 6 h (TLC indicated full conversion). Silica gel (3 g) was added to the reaction mixture and all volatiles were removed under high vacuum. The residue was purified by flash column chromatography (dichloromethane-methanol-7M NH_3 in methanol, 85:15:2), and then by reversed phase flash column chromatography (water-methanol-7 M NH_3 in methanol, 10:90:2, Biotage, column: 12 g). The product **SY-351**was obtained as a white solid (82 mg, 51%).

^{1}H NMR (500 MHz, dimethylsulfoxide-d_6) δ (ppm) 11.11 (br s, 2H), 8.87 (d, J = 2.5 Hz, 1H),

8.73 – 8.56 (m, 1H), 8.53 – 8.42 (m, 2H), 8.29 – 8.23 (m, 1H), 8.22 (d, *J* = 2.5 Hz, 1H), 8.01 (d, *J* = 8.6 Hz, 1H), 7.51 (br s, 1H), 7.29 – 7.23 (m, 1H), 7.23 – 7.08 (m, 2H), 6.80 (dt, *J* = 15.4, 5.8 Hz, 1H), 6.29 (dt, *J* = 15.5, 1.8 Hz, 1H), 4.09 – 3.82 (m, 2H), 3.07 (dd, *J* = 5.8, 1.7 Hz, 2H), 2.26 – 2.19 (m, 1H), 2.18 (s, 6H), 2.07 – 1.96 (m, 1H), 1.89 – 1.76 (m, 2H), 1.60 – 1.49 (m, 1H), 1.48 – 1.36 (m, 2H), 1.33 – 1.26 (m, 1H).

13C NMR (126 MHz, dimethylsulfoxide-d_6) δ (ppm) 163.85, 162.54, 159.63, 144.71, 142.86, 138.98, 138.13, 136.06, 130.88, 126.49, 126.39, 125.06, 122.43, 122.26, 120.63, 111.92, 59.64, 49.31, 47.24, 45.12, 38.62, 31.58, 31.47, 23.09.

HRMS (APCI): calcd. for $C_{30}H_{34}ClN_8O_2$ $[M+H]^+$ 573.2488, found 573.2486.

^{1}H (500 MHz) and ^{13}C NMR (126 MHz) spectra of **4** in dimethylsulfoxide-d_6

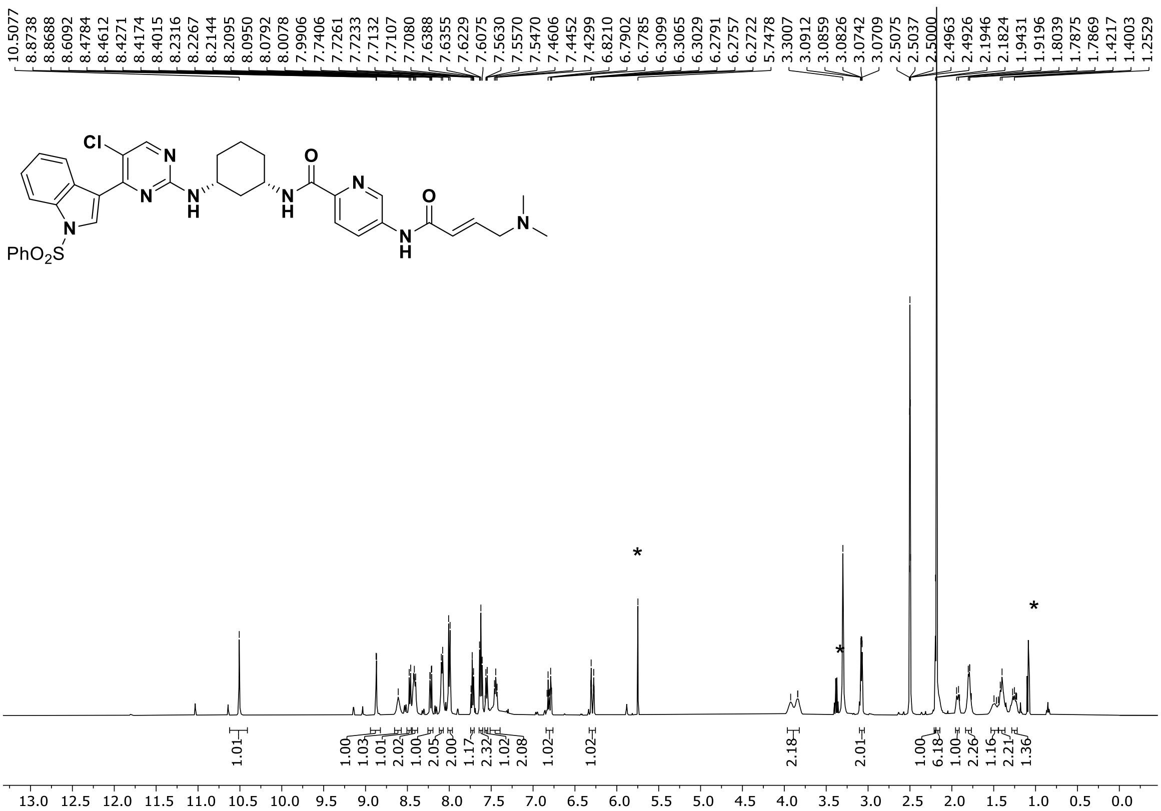

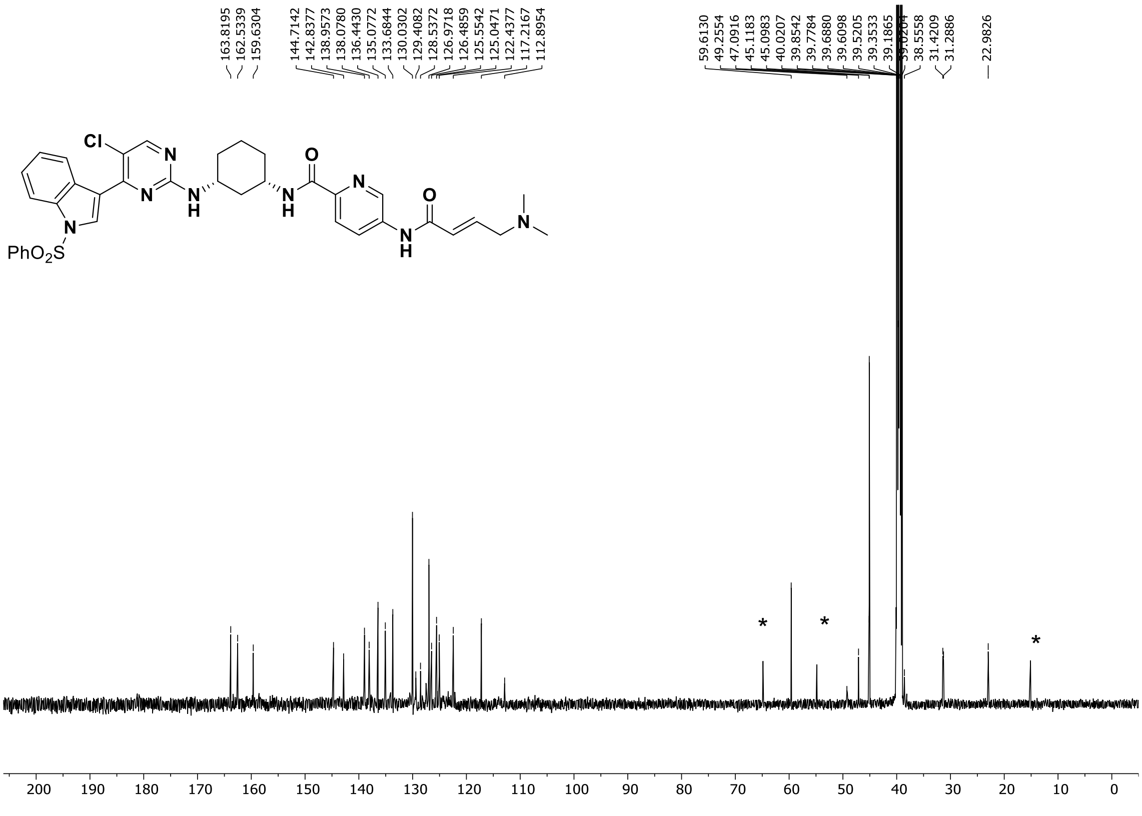

^{1}H-^{13}C *J*-edited HSQC and ^{1}H-^{13}C HMBC NMR spectra of **4** in dimethylsulfoxide-d_6

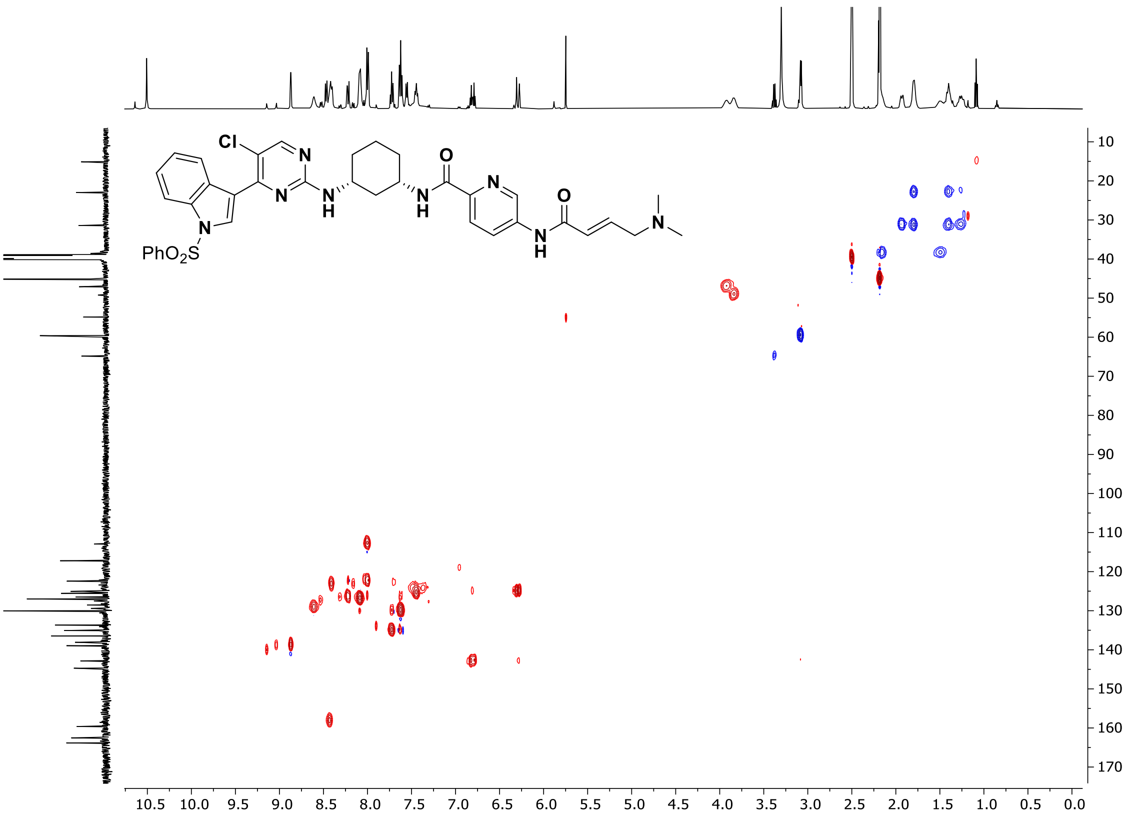

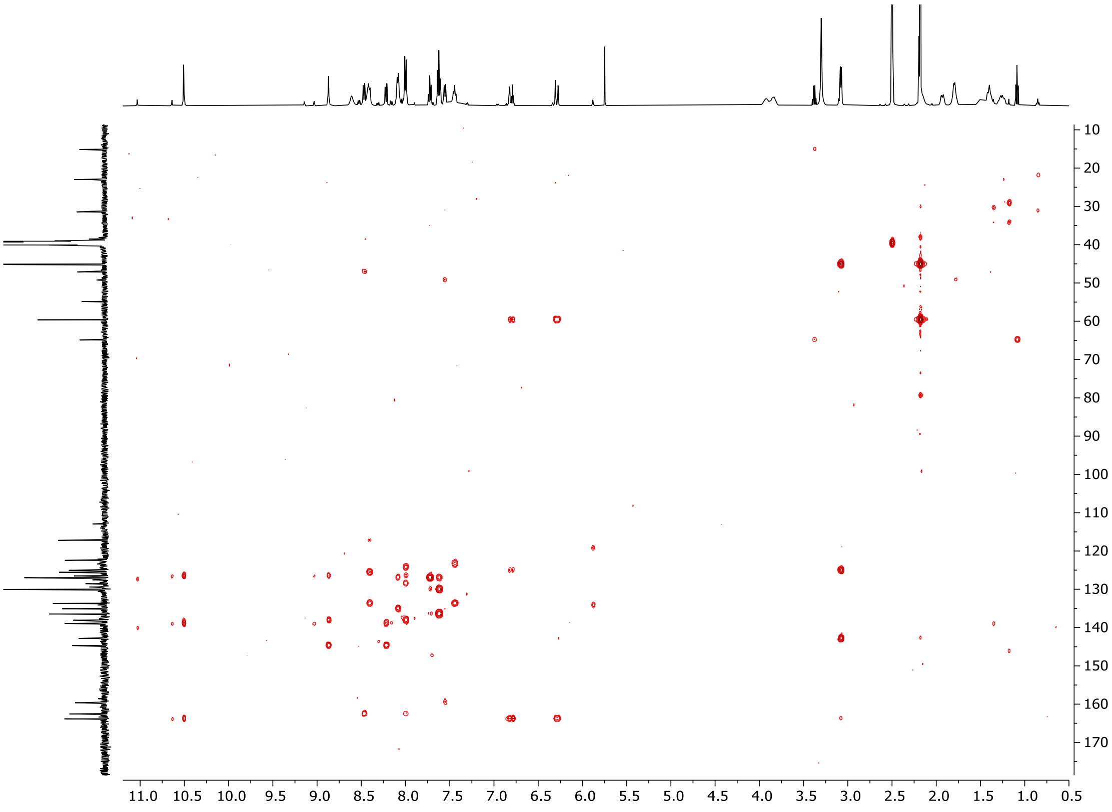

HRMS spectrum of **4**

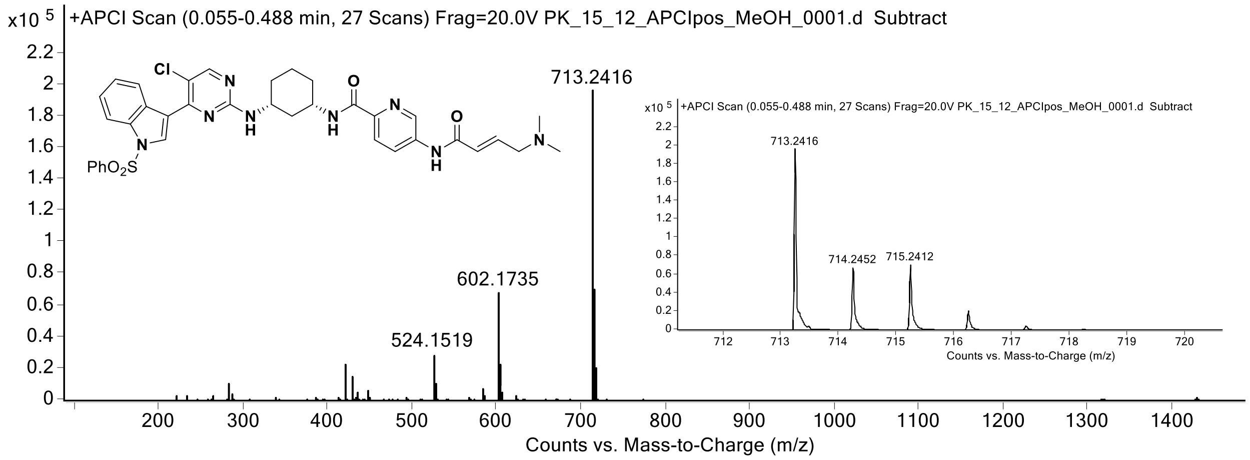

^{1}H (500 MHz) and ^{13}C NMR (126 MHz) spectra of **SY-351** in dimethylsulfoxide-d_6

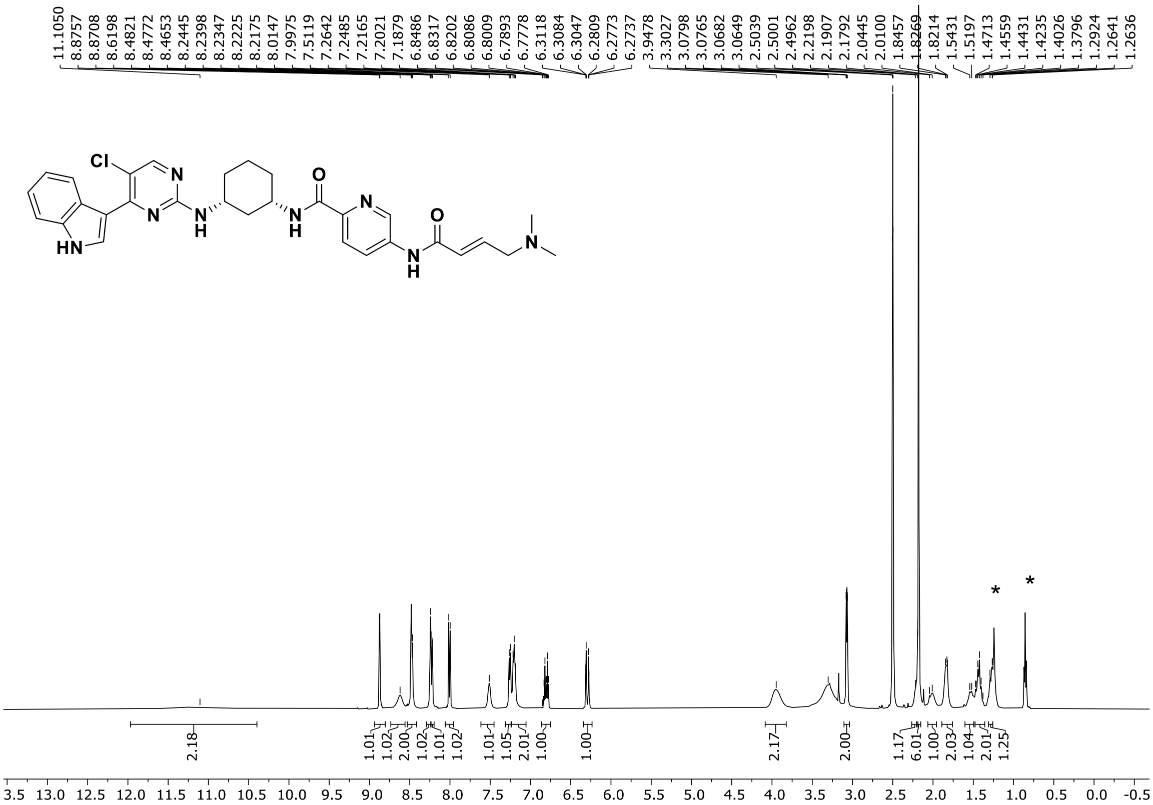

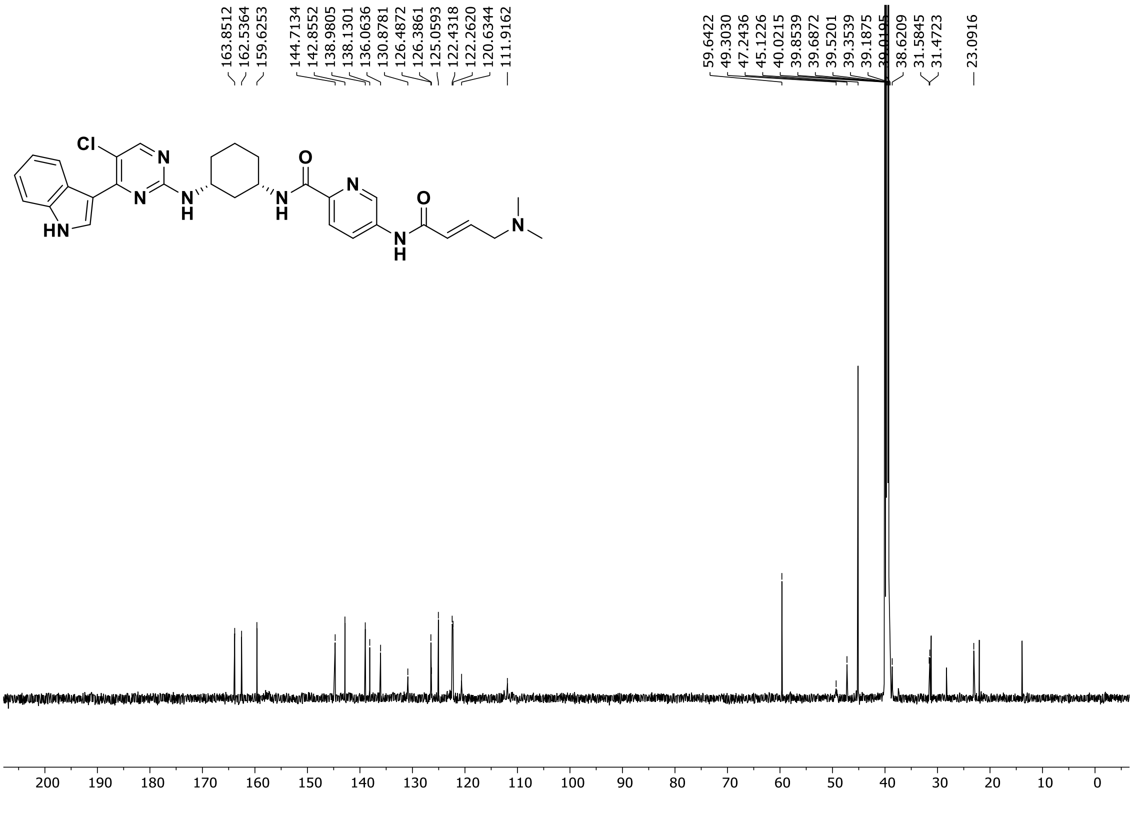

^{1}H-^{13}C *J*-edited HSQC and ^{1}H-^{13}C HMBC NMR spectra of **SY-351** in dimethylsulfoxide-d_6

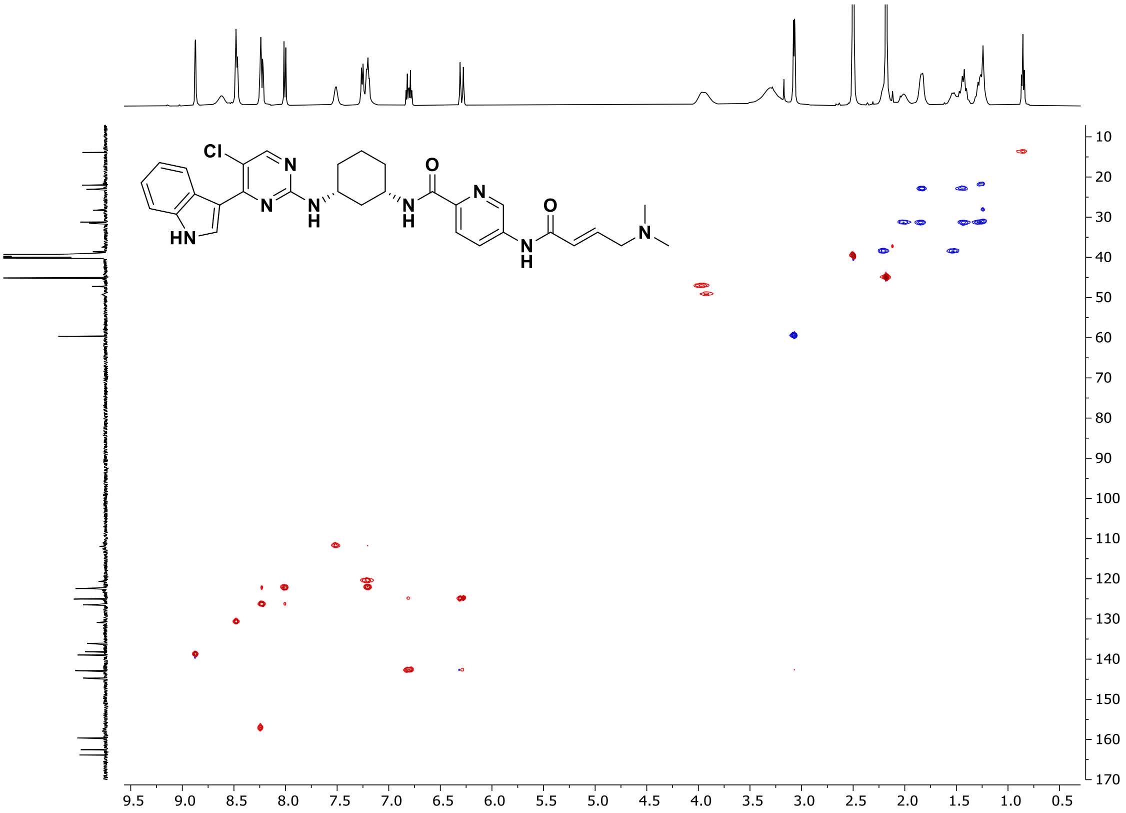

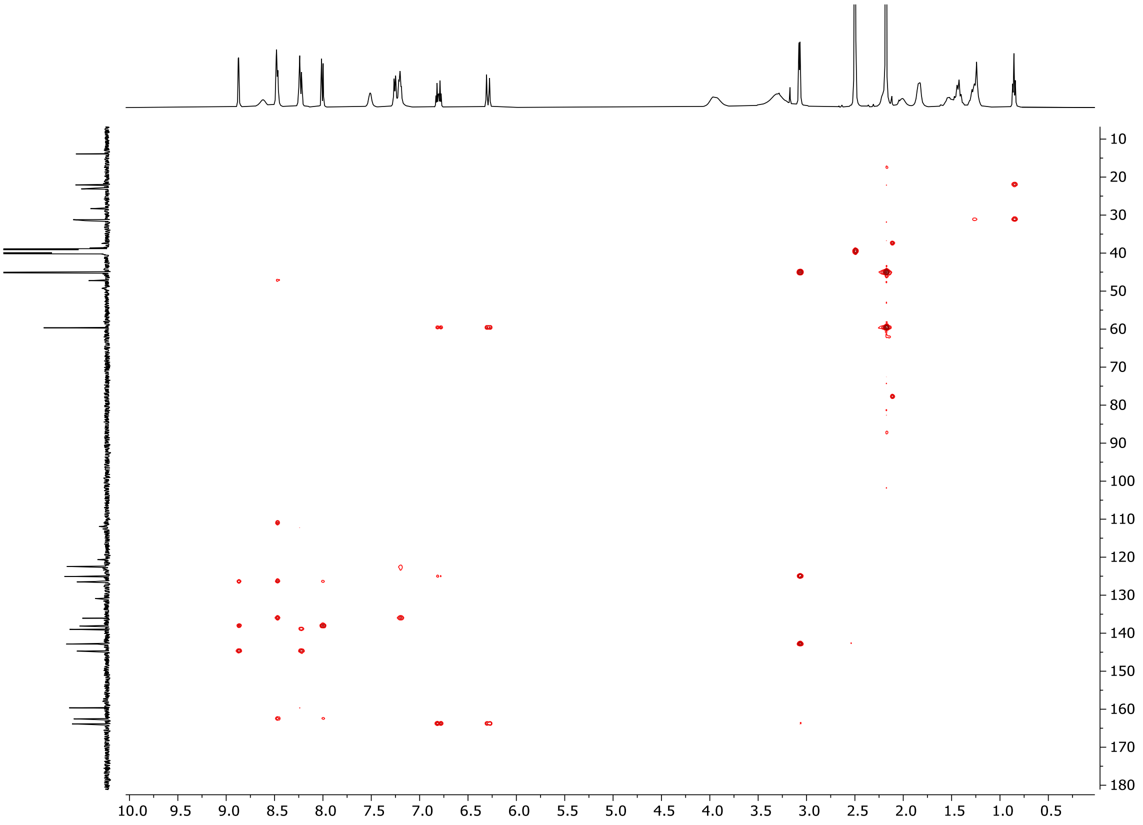

HRMS spectrum of **SY-351**

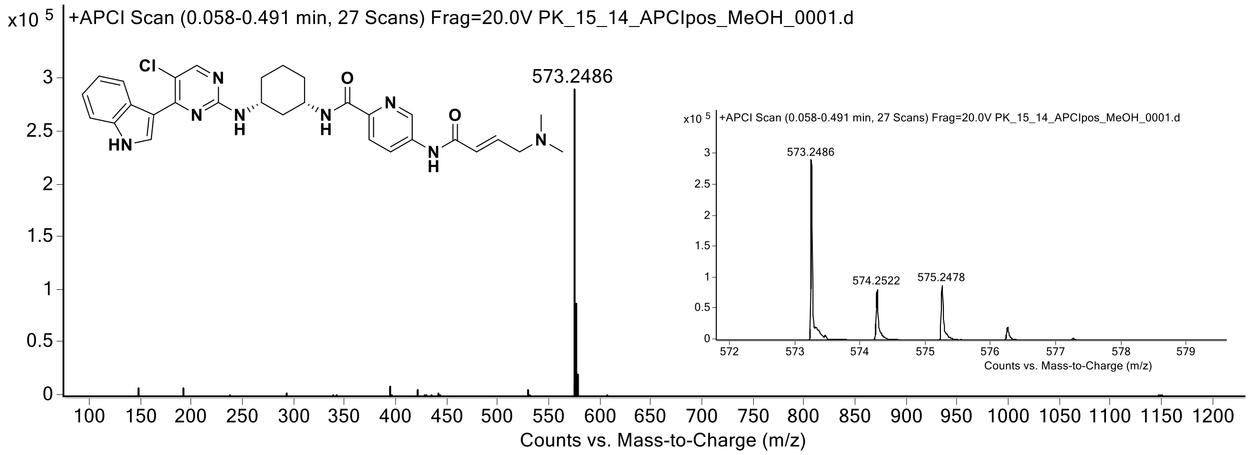

SUPPLEMENTARY REFERENCES

1. Hu, S. *et al.* Discovery and Characterization of SY-1365, a Selective, Covalent Inhibitor of CDK7. *Cancer Res* **79**, 3479-3491 (2019).
